# Enhancing anti-tumor immunity through intratumoral combination therapy with amphiphilic conjugates of oxaliplatin and imidazoquinoline TLR7/8 agonist†

**DOI:** 10.1039/d5ra00163c

**Published:** 2025-04-14

**Authors:** Hanne Verschuere, Sabah Kasmi, Lutz Nuhn, Sènan Mickaël D'Almeida, Qiwen Zhu, Zifu Zhong, Sandy Adjemian, Benoit Louage, Jana De Vrieze, Haijun Yu, Bruno G. De Geest, Peter Vandenabeele

**Affiliations:** a Cell Death and Inflammation Unit, VIB Center for Inflammation Research Ghent Belgium peter.vandenabeele@irc.vib-ugent.be; b Department of Biomedical Molecular Biology (DBMB), Ghent University Belgium; c Cancer Research Institute Ghent (CRIG), Ghent University Belgium br.degeest@ugent.be; d Department of Pharmaceutics, Ghent University Ghent Belgium; e Department of Chemistry and Pharmacy, Julius-Maximilians-Universität Würzburg Würzburg Germany; f CyTOF Flow Cytometry Core Facility, École Polytechnique Fédérale de Lausanne (EPFL) Lausanne Switzerland; g State Key Laboratory of Drug Research & Center of Pharmaceutics, Shanghai Institute of Materia Medica, Chinese Academy of Sciences Shanghai 201203 China; h Methusalem Program, Ghent University Belgium

## Abstract

The efficacy of conventional chemotherapy does not only rely on the cytotoxic action of the drug compound itself. Indeed, proper drug-induced immunogenic cell death (ICD) can stimulate immunosurveillance and mount a systemic anti-tumor response. We aimed to further amplify the therapeutic activity of oxaliplatin (OxPt) chemotherapy-induced ICD by combining this with an imidazoquinoline (IMDQ) TLR7/8 agonist. We hypothesized that innate immune activation by TLR7/8 activation primes the immune system against tumor neoantigens, thereby mounting tumor-specific T cell responses that contribute to killing primary tumor cells and distal metastases. To this end, we initially synthesized a covalent conjugate of OxPt, an imidazoquinoline TLR7/8 agonist (*i.e.*, IMDQ), and an alkyl lipid. We hypothesized that such a lipidated conjugate would, upon intratumoral injection, increase the residence time in the tumor and reduce systemic dissemination and, hence, off-target toxicity. Whereas combination therapy with OxPt and IMDQ in native form improved, relative to single treatment, the anti-tumor efficacy against the primary treated tumor and a secondary distal tumor, this was not the case for OxPt–IMDQ-lipid conjugate therapy. We then altered the molecular design of the combination therapy and synthesized amphiphilic OxPt and IMDQ conjugates, comprising a cholesteryl motif and a hydrophilic poly(ethylene glycol) (PEG) chain. Intratumoral combination therapy with OxPt-PEG-cholesteryl and IMDQ-PEG-cholesteryl reduced, compared to native drug compounds, systemic innate inflammatory responses, and more efficiently eradicated primary and distal tumors. Furthermore, we found that combination therapy with OxPt-PEG-cholesteryl and IMDQ-PEG-cholesteryl induced antigen-specific anti-tumor responses and high infiltration levels of CD8+ T cells into the tumor.

## Abstract 2

Combining a cytotoxic drug with an immune-activating drug eradicates tumors.

## Graphical abstract

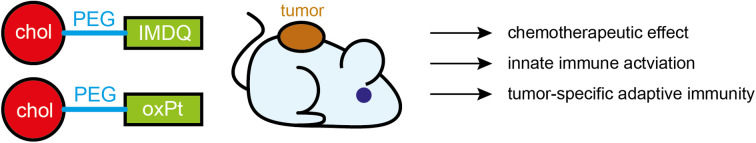

## Introduction

Chemotherapy is widely used for treating various cancers.^1^ Despite its efficacy, conventional chemotherapy often fails to provide long-term protection against relapse and metastasis. Interestingly, certain chemotherapeutics such as oxaliplatin, mitoxantrone, and doxorubicin can trigger an adaptive immune response by inducing immunogenic cell death (ICD).^2,3^ ICD enhances anti-tumor immunity through adjuvanticity, inflammation, and antigenicity.^4,5^ During ICD, damage-associated molecular patterns (DAMPs), cytokines, and chemokines are released into the tumor microenvironment, promoting the recruitment and maturation of dendritic cells (DCs).^6,7^ These DCs then phagocytize dying cancer cells and present tumor-associated antigens (TAAs) on their surface, leading to T cell expansion and a tumor-specific CD8+ T cell response.^8,9^ However, cancer cells often develop mechanisms to evade immune detection and destruction, allowing tumors to grow and spread.^10^ In recent years, immunotherapy with immune checkpoint inhibitors (such as anti-PD-L1, anti-CTLA-4, and anti-PD-1 monoclonal antibodies) has emerged as a promising strategy to counteract immune escape.^11^ Unfortunately, these therapies are not universally effective, often due to a lack of antigen-specific cytotoxic CD8+ T cells infiltrating the tumor.^12^

To enhance the efficacy of these treatments, activating innate immune receptors like Toll-like receptors 7/8 (TLR7/8) in antigen-presenting cells within the tumor microenvironment or draining lymph nodes is a promising approach.^13^ Imidazoquinolines, potent small molecule agonists of TLR7/8, have been shown to induce IL-12 and type I interferon secretion by APCs,^14^ thereby priming the adaptive immune system to mount potent anti-viral and anti-tumor responses.^15–17^ By integrating ICD-inducing therapies with TLR7/8 stimulation, it is possible to convert ‘cold’, poorly immunogenic tumors into ‘hot’ tumors with robust inflammatory profiles.^18,19^ Combining ICD-inducing chemotherapeutics like oxaliplatin with DC-activators such as IMDQ could potentially eradicate tumors by creating a hot tumor microenvironment and eliciting a strong adaptive anti-tumor response. This combination strategy may offer a synergistic clinical benefit and improve the overall effectiveness of cancer immunotherapy.^20–27^

However, small molecule drugs exhibit rapid dissemination throughout the body and are prone to systemic off-target toxicity.^28,29^

Lipid-drug conjugates have gained significant interest in drug delivery for enhancing the therapeutic efficacy of various drugs. They offer several advantages, including improved bioavailability, enhanced targeting of specific cells and reduced off-target toxicity. Lipid-drug conjugates are being explored for diverse applications, particularly in oncology, infectious diseases and auto-immune disorders. Current research focuses on the rational design of the lipid moieties, along with careful selection of the linkers.^30,31^

Here we report on lipophilic and amphiphilic OxPt and IMDQ conjugates by chemically modifying the axial positions of OxPt to increase the retention time of the drugs within the tumor microenvironment upon intratumoral injection. Notably, conjugation at the axial Pt positions alters the oxidation state of Pt from II to IV.^32^ The cytotoxic mechanism of action of Pt drugs relies on inhibiting DNA synthesis by forming covalent bonds with nitrogen atoms in the purine bases (adenine and guanine) of DNA. Pt(iv) is kinetically inert in this reaction and must be reduced, *e.g.*, by intracellular glutathione, to Pt(ii) to become biologically active.^33,34^ Hence, chemical conjugation at the axial Pt positions in OxPt transforms OxPt into an oxidated prodrug.^34–37^ Here, we demonstrate that intratumoral combination therapy with PEG-cholesteryl conjugates of OxPt and IMDQ induces local innate immune activation in the tumor microenvironment and mounts tumor antigen-specific adaptive T cell responses.

## Results

### Therapeutic synergism between oxaliplatin and IMDQ completely eradicates primary tumors

**Fig. 1:**
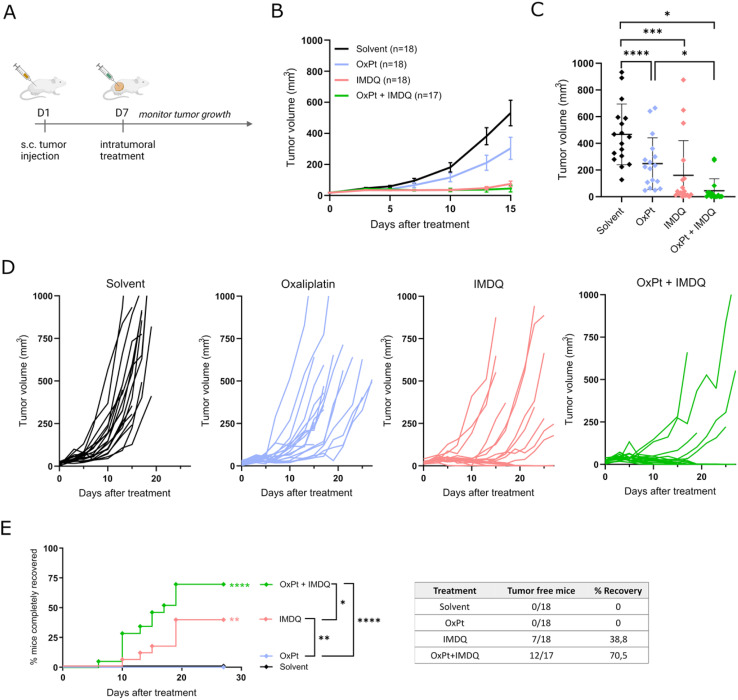
Oxaliplatin + IMDQ combination therapy has a synergistic effect on tumor eradication. (A) Schematic representation of the experimental setup. Balb/c mice were injected subcutaneous with CT26 cells to induce tumor formation. After 7 days the tumor was treated intratumorally (IT) (solvent, oxaliplatin (OxPt), imidazoquinoline (IMDQ) *n* = 18; OxPt + IMDQ; *n* = 17 for all subfigures; pool of 3 independent experimental repeats). (B) Volume of the subcutaneous tumors starting from the moment of IT treatment. Data represented as mean ± SD. (C) Tumor volume at day 15 after treatment. Data represented as mean ± SD, dots depict different mice. All significant comparisons are indicated. (D) Tumor growth progression over time starting at the moment of IT treatment, each mice is visualized individually until the moment of euthanasia. Animals were euthanized when tumor size exceeded 1000 mm^3^ or upon necrosis in the tumor. (E) Complete recovery from tumor. When tumor size decreased below 25 mm^3^ or only scar tissue remained, this was considered complete recovery. Asterisks next to the curve indicate significant differences with solvent control. Statistic in the legend compares the different treatments. Table gives the exact numbers that are depicted in the graph. *p < 0.05, **p < 0.01, ***p < 0.001, ****p < 0.0001.

We evaluated the therapeutic synergism between intratumoral injection of oxaliplatin (OxPt) and the imidazoquinoline TLR7/8 agonist 1-(4-(aminomethyl)benzyl)-2-butyl-1*H*-imidazo[4,5-*c*]quinolin-4-amine (IMDQ) in a syngeneic CT26 mouse tumor model. To this end, Balb/c mice were injected subcutaneously with 5 × 10^5^ CT26 mouse colon cancer cells to initiate tumor formation (Fig. 1A). After 7 days, treatment was administered *via* intratumoral (IT) injection of either OxPt, IMDQ, a combination (OxPt + IMDQ), or a solvent vehicle control. OxPt monotherapy reduced the tumor growth rate compared to the solvent vehicle control, but only to a modest extent and did not fully eradicate the tumor (Fig. 1B–D). IMDQ monotherapy exerted a strong effect on decreasing tumor growth, confirming previous reports. OxPt + IMDQ combination therapy further reduced the tumor growth rate (Fig. 1C). More importantly, the fraction of completely responding tumor-free animals was the highest in the cohort receiving OxPt + IMDQ combination therapy, resulting in 70% of the mice being completely cured, compared to 39% by IMDQ treatment and none by OxPt treatment (Fig. 1E).

### Synthesis and *in vitro* evaluation of IMDQ–OxPt–C18

Based on the beneficial therapeutic response to OxPt + IMDQ combination therapy, we explored whether chemical conjugation of OxPt and IMDQ could enhance the therapeutic response even further. The rationale for this was fostered by the hypothesis that co-localization of tumor antigens from dying cells and IMDQ in the tumor microenvironment would lead to an improved antigen-specific immune response and, hence, an improved abscopal effect on distal tumors. We further reasoned that additional conjugation of a lipid tail to the OxPt–IMDQ conjugate would, upon IT injection, limit diffusion of the drug molecules into systemic circulation, and thereby limit systemic off-target cytotoxicity and innate immune activation.

A C18 alkyl lipid was first conjugated to the axial position of OxPt, resulting in an asymmetric OxPt–C18 prodrug. Several studies have reported that more hydrophobic Pt prodrugs exhibit higher cytotoxicity through increased cellular uptake. IMDQ was then conjugated to the remaining axial position of OxPt (Fig. S15A†). IMDQ–OxPt–C18 was synthesized based on literature procedures for platinum modification. In the first step, OxPt was oxidized in aqueous medium in the presence of hydrogen peroxide at room temperature for 24 hours, resulting in the substitution of two hydroxo ligands onto the axial positions of the Pt atom. The *trans*-dihydroxo complexes were then reacted by nucleophilic substitution with an equimolar amount of succinic anhydride, resulting in a mono-carboxylate conjugate. The remaining hydroxyl group was then reacted with octadecyl isocyanate, forming a carbamate linkage, resulting in OxPt–C18. Finally, IMDQ was conjugated to OxPt by amide bond formation between the aliphatic primary amine of IMDQ and the succinate carboxylic acid group of OxPt–C18, yielding IMDQ–OxPt–C18. All intermediates and the final compound were obtained in high yields ranging from 75 to 95% and fully characterized by ESI-MS and NMR spectroscopy (Fig. S1–S12, ESI†).

Uptake of all Pt (pro)drugs (*i.e.*, OxPt, OxPt–C18, and IMDQ–OxPt–C18) by CT26 cells *in vitro* was assessed using CyTOF mass cytometry analysis. A dose- and time-dependent increase in Pt-positive cells was observed for the different prodrugs (Fig. S15B†). OxPt was internalized faster than the more hydrophobic conjugates, featuring higher percentages of Pt + cells after 4 hours of treatment. However, after 24 hours, higher percentages of Pt + cells at lower concentrations were observed for the Pt prodrugs compared to OxPt. In addition, the intracellular Pt content, assessed by measuring the Pt-ion intensity per cell (Fig. S15C†), reached much higher levels when cells were treated with the more hydrophobic Pt prodrugs. Notably, after 24 hours, cells treated with IMDQ–OxPt–C18 featured a 100-fold increase in Pt content compared to cells treated with native OxPt. This indicates that chemical conjugation alters the kinetics and extent of the cellular Pt uptake.

Next, we evaluated the TLR agonistic activity of the IMDQ–OxPt–C18 prodrug. Hereto, Raw-Blue macrophage NF-κB-reporter cells were pulsed for 24 hours with either soluble IMDQ, OxPt–C18, IMDQ–OxPt–C18, or a combination of native OxPt and IMDQ. IMDQ–OxPt–C18 treatment triggered TLR activation *in vitro*, whereas both oxaliplatin and OxPt–C18 did not induce a response (Fig. S15D†). IMDQ–OxPt–C18 was less potent than native IMDQ. Such reduction in potency of chemically conjugated IMDQ is in line with previous reports by us and others.

Subsequently, we assessed the *in vitro* cytotoxicity of the Pt (pro)drugs on the colon cancer cell line CT26 (Fig. S15E†). Cells were incubated with a concentration range of Pt (pro)drugs, and native IMDQ and OxPt. Cell viability was evaluated at three consecutive time points, *i.e.*, after 24, 48, and 96 hours of treatment, to assess the time-dependence of the cytotoxicity. This choice was fostered by the consideration that the Pt prodrugs require reduction to release Pt in a cytotoxic active form. The IC50 values of the Pt prodrugs are 2–3 fold lower than the IC50 of OxPt (Fig. S15E and Table S1 (ESI)†), indicating that a higher cellular uptake of the Pt prodrugs correlates with increased potency.

Finally, we investigated the innate activation of IMDQ and IMDQ–OxPt–C18 *in vivo*. Hereto, a transgenic IFN-β-luciferase reporter mouse model, with a reporter gene linked to the expression of type I interferon, IFNβ, was used. Native IMDQ and IMDQ–OxPt–C18 were injected subcutaneously in the flank of the mice, followed by non-invasive whole-body luminescence imaging at different time points, *i.e.*, 4 hours, 24 hours, and 48 hours. In line with our earlier reports, subcutaneous injection of native IMDQ in the flank of the mice induced a rapid systemic bioluminescence response. By contrast, IMDQ–OxPt–C18 elicited a local immune activation at the site of injection without provoking systemic immune activation (Fig. S15F and G†).

### IMDQ–OxPt–C18 is less potent in tumor eradication than combination therapy with unformulated OxPt and IMDQ

The therapeutic anti-tumor effect of IMDQ–OxPt–C18 was again evaluated in a syngeneic CT26 mouse model, comparing IT treatment of OxPt + IMDQ with IMDQ–OxPt–C18 as combination therapy and IMDQ or OxPt as a monotherapy (Fig. S16A†). IMDQ–OxPt–C18 reduced the tumor growth rate, albeit less efficiently than the combination therapy with OxPt and IMDQ (Fig. S16B–D†). Treatment with the IMDQ–OxPt–C18 prodrug, in contrast to the combination therapy, did not result in complete recovery (Fig. S16E†). Thus, chemical conjugation of OxPt and IMDQ could not improve the therapeutic anti-tumor activity compared to combination therapy with native OxPt and IMDQ, but instead decreased the anti-tumor response.

Additionally, mice were challenged one week post-treatment with an injection of live CT26 cells in the opposite flank to assess whether IT treatment in the primary tumor could prevent the growth of a distal tumor (Fig. S16A†). Such prevention requires activation of the adaptive immune system and T cell priming. Strikingly, whereas treatment with native IMDQ or the OxPt + IMDQ combination therapy provided protection against a distal tumor, this effect was not observed upon treatment with IMDQ–OxPt–C18 (Fig. S16F†). This suggests that conjugation to either oxaliplatin or stearyl may prevent or reduce the effect of IMDQ.

### Combination therapy with amphiphilic OxPt and IMDQ conjugates is more potent than unformualted soluble OxPt + IMDQ

**Fig. 2:**
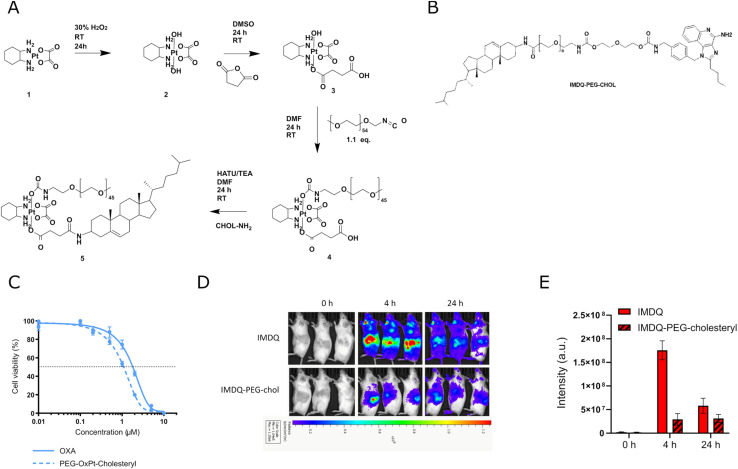
Synthesis, *in vitro* evaluation and bioluminescence imaging of amphiphilic platinum prodrug. (A) Synthesis route of amphiphilic platinum prodrug. (B) Chemical structure of IMDQ-PEG-cholesteryl conjugate. (C) Cytotoxicity profiles of oxaliplatin and amphiphilic platinum prodrug (OxPt-PEG-cholesteryl) after 96 hours on CT26-cells by MTT assay (*n* = 5, mean + SD). (D) *In vivo* innate immune activation of IMDQ and IMDQ-PEG-cholesteryl conjugate. Body distribution of IFN-β expression after subcutaneous injection of soluble IMDQ and amphiphilic platinum prodrug in the flank of luciferase reporter mice after 4 h and 24 h (*n* = 3). (E) Quantification oftotal luciferase luminescence intensity.

We reasoned that the limited solubility of the IMDQ–OxPt–C18 conjugate could be responsible for its limited therapeutic activity *in vivo*. We therefore decided to synthesize an amphiphilic Pt prodrug (Fig. 2A) to mitigate the solubility issue. Furthermore, amphiphile design has emerged as powerfull startegy for local immune activation. Recently, we reported on the conjugation of IMDQ to a cholesteryl-poly(ethylene glycol), cholesteryl-PEG, amphiphile. The resulting conjugate, IMDQ-PEG-cholesteryl (Fig. 2B), was well soluble in aqueous medium and elicited local innate immune activation upon injection. Fostered by these findings, we applied a similar molecular design to synthesize an amphiphilic OxPt-PEG-cholesteryl conjugate. As combination treatment with OxPt and IMDQ had a greater therapeutic benefit than an OxPt-IMDQ conjugate, we endeavored into testing combination therapy of OxPt-PEG-cholesteryl and IMDQ-PEG-cholesteryl.

A 2 kDa PEG chain and cholesterylamine were conjugated to each axial position of OxPt (Fig. 2A). OxPt-PEG-cholesteryl was synthesized according to previously described procedures and characterized by ^1^H and DOSY NMR spectroscopy (Fig. S13, ESI†). Accordingly, the free hydroxyl group of the mono-succinate Pt intermediate was reacted with PEG-isocyanate, forming a carbamate bond. Next, cholesterylamine was conjugated by amide bond formation to the succinate carboxylic acid moiety, resulting in OxPt-PEG-cholesteryl. The *in vitro* cytotoxicity of the OxPt-PEG-cholesteryl conjugate was assessed on CT26 cells by MTT assay (Fig. 2C). In contrast to the previous, hydrophobic, IMDQ–OxPt–C18 conjugate, after 96 hours of incubation, the amphiphilic OxPt-PEG-cholesteryl conjugate featured a similar dose-dependent cytotoxicity as native OxPt.

We tested innate immune activation of the IMDQ-PEG-cholesteryl conjugate upon IT injection in CT26 tumor-bearing IFN-β-luciferase reporter mice (Fig. 2D–E). Seven days post-tumor implantation, IMDQ and IMDQ-PEG-cholesteryl were injected IT, followed by bioluminescence imaging. In line with our previous reports, IMDQ-PEG-cholesteryl triggered localized innate immune activation with a strong reduction in systemic innate immune activation compared to native IMDQ.

**Fig. 3:**
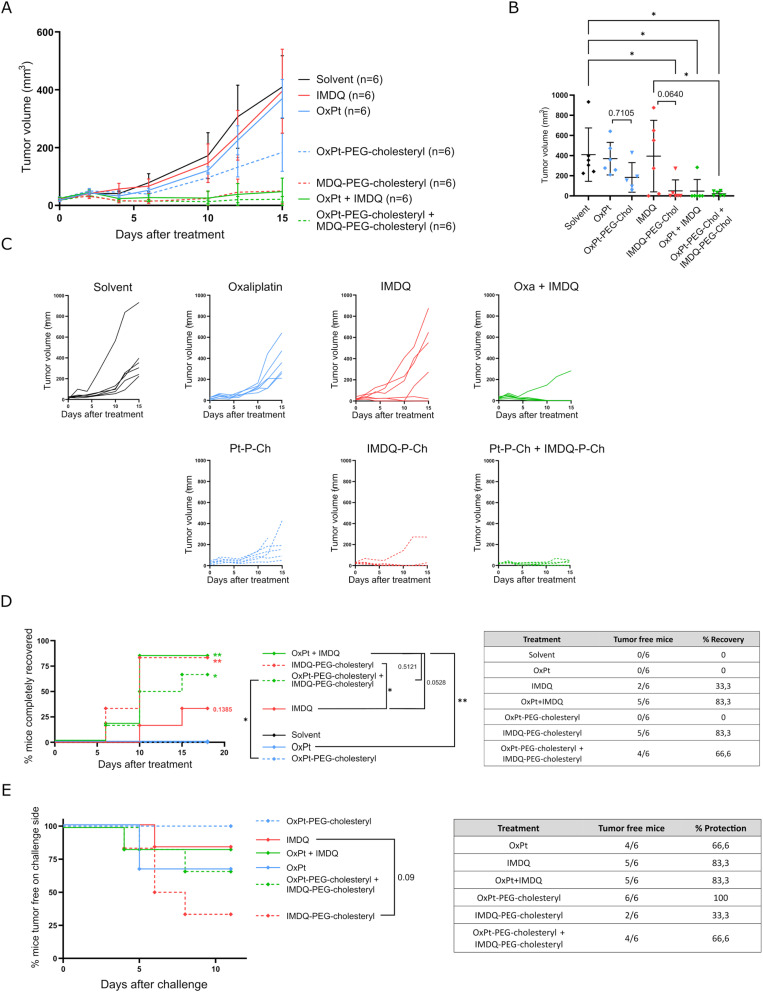
PEG-Chol conjugation increases the therapeutic efficacy of oxaliplatin and IMDQ. (A) Mice were injected subcutaneous with CT26 cells to start tumor formation. After 7 days tumors were treated intratumorally. Another 7 days later CT26 cells were injected in the opposite flank to induce a challenge tumor. Volume of the subcutaneous tumors was measured from the moment of treatment until day 15. Data represented as mean ± SD (*n* = 6 for all conditions in all subfigures). (B) Tumor volume at day 15 after treatment. Data represented as mean ± SD, dots depict different mice. All significant comparisons are indicated. (C) Complete recovery from tumor. Asterisks next to the curve indicate significant differences with solvent. Statistics in the legend compare different treatments. Table gives exact numbers from the graph. (D) Tumor growth progression over time starting at the moment of treatment, each mice is visualized individually. (E) Registration of growth onset of the challenge tumors. Graph starting from the moment of challenge, on day 14 of the experiment. Mice that did not develop a challenge tumor were considered tumor-free on the challenge side. *p < 0.05, 847 **p < 0.01, ***p < 0.001, ****p < 0.0001.

The therapeutic *in vivo* efficiency of the amphiphilic conjugates was evaluated in an identical setting as described above (*vide supra*, Fig. S17A†). A comparison was made between OxPt *versus* OxPt-PEG-cholesteryl, IMDQ *vs.* IMDQ-PEG-cholesteryl, and OxPt + IMDQ *vs.* OxPt-PEG-cholesteryl + IMDQ-PEG-cholesteryl. Amphiphilic OxPt and IMDQ conjugates were more efficient in decreasing the tumor growth rate compared to native compounds (Fig. 3A–D). The amphiphilic IMDQ conjugate provoked a strong anti-tumor effect, resulting in complete tumor eradication. On the other hand, single OxPt-PEG-cholesteryl treatment only reduced the tumor growth rate. Combination IT therapy with the amphiphilic conjugates, OxPt-PEG-cholesteryl and IMDQ-PEG-cholesteryl, induced a similar therapeutic effect compared to combination therapy with the native compounds, which was already very effective (Fig. 3C).

We also assessed whether an adaptive anti-tumor immune response was mounted after IT injection of the amphiphilic conjugates. Remarkably, IMDQ-PEG-cholesteryl induced lower protection against a challenge with live tumor cells compared to treatment with native IMDQ (Fig. 3E). Besides IMDQ-PEG-cholesteryl, all treatments resulted in a protection between 67% and 83%. IT treatment of the primary tumor with OxPt-PEG-cholesteryl even conferred full protection against a tumor challenge, thereby suggesting a robust induction of adaptive immunity.

### Combination therapy with amphiphilic OxPt and IMDQ conjugates increases activation of CD8+ T cells

**Fig. 4:**
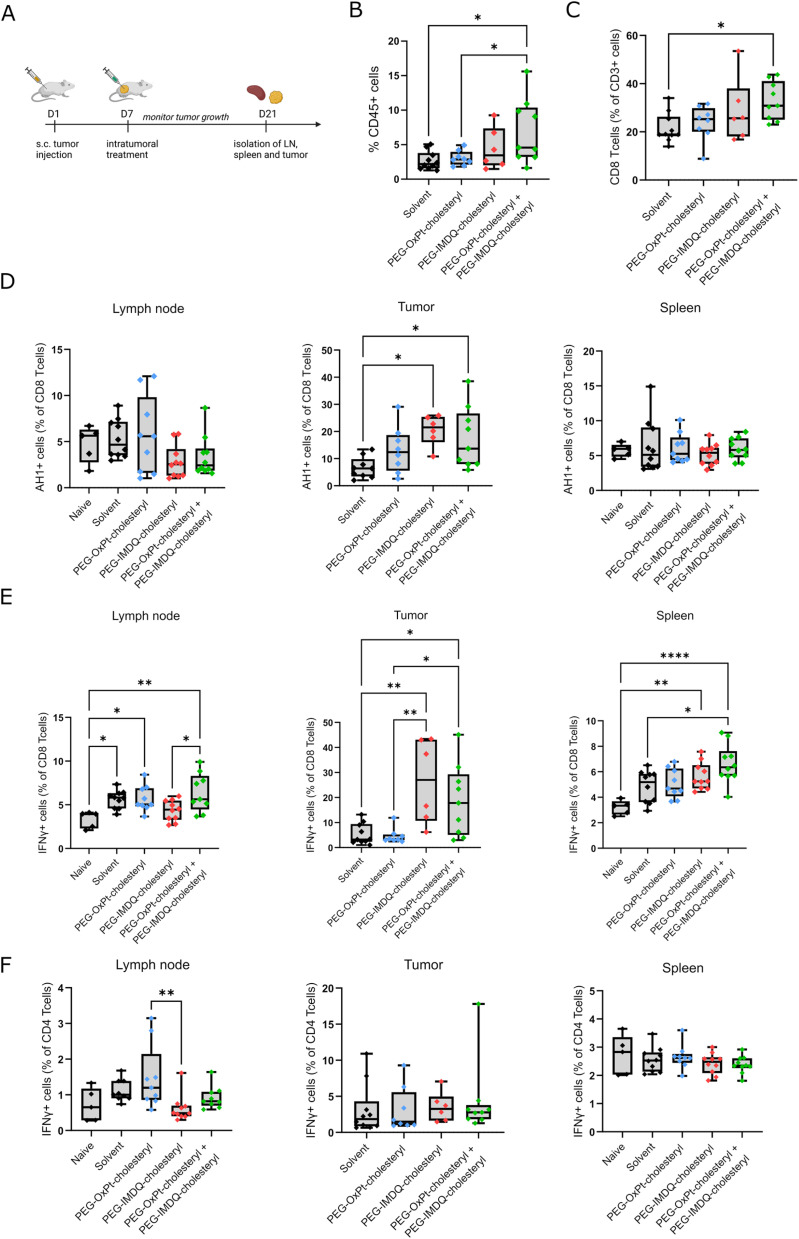
Analysis of the immune response after PEG-Chol treatments. (A) Schematic representation of the experiment setup. Balb/c mice were inoculated subcutaneous with CT26 cells to start tumor formation, after 7 days tumors were treated intratumorally. 14 Days post-treatment spleen, tumor draining lymph node (tdLN) and tumor tissue were isolated (data is pooled from 3 independent experimental repeats). (B) Percentage of leukocytes and (C) CD8 specific lymphocytes in the tumor tissue after different IT treatments. Each dot represent a tumor from a different mouse, all significant comparisons are indicated (solvent *n* = 10, OxPt-PEG-cholesteryl *n* = 8, IMDQ-PEG-cholesteryl *n* = 6, OxPt-PEG-cholesteryl + IMDQ-PEG-cholesteryl *n* = 9). (D) Post treatment percentage of CD8+ lymphocytes from spleen, tdLN and tumor with AH1-antigen-specific MHCI expression. (E) IFN-γ-producingg cells, percentage of CD8+ lymphocyte subpopulation and (F) CD4+ lymphocyte subpopulation in samples from spleen, tdLN and tumor after different treatments. Each dot depicts a separate mouse, all significant comparisons are indicated (spleen and lymph node samples: solvent, IMDQ-PEG-cholesteryl, OxPt-PEG-cholesteryl + IMDQ-PEG-cholesteryl *n* = 10; OxPt-PEG-cholesteryl *n* = 9. Tumor samples: solvent *n* = 10, OxPt-PEG-cholesteryl *n* = 8, IMDQ-PEG-cholesteryl *n* = 6, OxPt-PEG-cholesteryl + IMDQ-PEG-cholesteryl *n* = 9). *p < 0.05, **p < 0.01, ***p < 0.001, ****p < 0.0001.

To investigate whether IT injection of amphiphilic Pt- and IMDQ-conjugates mounts a tumor antigen-specific adaptive immune response, we assessed cross-priming and activation of cytotoxic T cells in the spleen, the tumor draining lymph node (tdLN, in this case the inguinal lymph node), and tumor tissue 14 days after IT treatment (Fig. 4A). Tumors were treated IT with OxPt-PEG-cholesteryl, IMDQ-PEG-cholesteryl, combination therapy, or a solvent vehicle control. All treatments successfully reduced the tumor growth rate compared to the solvent vehicle control, thereby confirming our previous observations (Fig. S17A and B,†*vide supra*Fig. 3A). Furthermore, we also dissected the tumor at the endpoint and determined its weight. Tumor weight within the different groups corresponded well with the measured differences in tumor size (Fig. S17B and C†). Of note, IT treatment with IMDQ-PEG-cholesteryl induced an increase in the size of the spleen and tdLN (Fig. S17D–F†). More efficacious treatments, *i.e.*, IMDQ-PEG-cholesteryl and OxPt-PEG-cholesteryl + IMDQ-PEG-cholesteryl, induced infiltration of a higher number of CD45+ cells into the tumor, as well as an increase in CD8+ T cell percentage of the total T cell population in the tumor (Fig. 4B and C).

CT26 cells contain a tumor-associated antigen, *i.e.*, GP70 processed to the AH1 epitope, that can be cross-presented by DCs to prime T cells. We then evaluated the capacity of the different treatments to induce an antigen-specific T cell response. Single-cell suspensions were prepared from the dissected spleen and tdLN and analyzed by flow cytometry with anti-AH1 tetramer staining (the gating strategy is depicted in Fig. S18†). The most effective treatments, *i.e.*, IMDQ-PEG-cholesteryl and OxPt-PEG-cholesteryl + IMDQ-PEG-cholesteryl, also elicited an increased AH1-specific anti-tumor response, demonstrated by an increase in the tumor infiltration of AH1+/CD8+ T cells. However, this AH1-specific T cell population was not present in the tdLN (Fig. 4D). OxPt-PEG-cholesteryl treatment, which only slows down tumor growth, induced a lower infiltration of AH1 antigen-specific T cells in the tumor (Fig. 4D and E). Accordingly, higher percentages of IFNγ-producing CD8+ T cells were detected in tumors treated with IMDQ-PEG-cholesteryl, but not in OxPt-PEG-cholesteryl treated tumors. This highlights the importance of DC activation in eliciting an effective anti-tumor response.

The tdLN of tumor-bearing mice features increased numbers of IFNγ-producing CD8+ T cells compared to naïve mice, yet these numbers do not further increase after treatment (Fig. 4E). IMDQ-PEG-cholesteryl treatment resulted in significantly lower activation of CD8+ T cells in the tdLN compared to combination therapy (Fig. 4E) and lower activation of CD4+ T cells compared to OxPt-PEG-cholesteryl (Fig. 4F). This decreased population of activated CD4+ T cells upon IMDQ-PEG-cholesteryl treatment might explain the previously observed lack of protection against a challenge tumor (Fig. 3E).

## Discussion

OxPt is a standard-of-care therapy in colorectal carcinoma patients. Although OxPt is an immunogenic chemotherapeutic drug, OxPt monotherapy often does not result in complete healing of the tumor due to additional immunosuppressive factors in the tumor microenvironment. In the present work, we show that, despite reducing the tumor growth rate, IT OxPt monotherapy is unable to mount complete tumor clearance. Additional innate immune activation by combination therapy with an imidazoquinoline TLR7/8 agonist greatly improves tumor eradication, thereby confirming similar studies. Our study differs from these in the sense that Hao *et al.*^21^ designed nanoparticles containing both imidazoquinoline and OxPt, while we used amphiphilic IMDQ- and Pt-conjugates that are soluble in aqueous medium. Liu *et al.*,^20^ on the other hand, established a similar subcutaneous tumor model as we used, but treatment was done intraperitoneally instead of intratumorally, and therefore a 6 times higher dose of OxPt was needed. Furthermore, resiquimod was used as a TLR7/8 agonist at a similar concentration as the imidazoquinoline analogue in our model.

In our original attempt to enhance the complementary anti-tumor mechanisms of oxaliplatin and IMDQ, we synthesized a hydrophobic prodrug comprising Pt being covalently bound to IMDQ and a C18 alkyl lipid (*i.e.*, OxPt–IMDQ–C18). Several studies have reported on the design of lipophilic Pt prodrugs by incorporating alkyl ligands as axial ligands. For example, Zheng *et al.*^37^ systematically varied the length of the aliphatic tail to tune cellular uptake and cytotoxicity of the Pt prodrug. However, despite efficient cellular uptake *in vitro*, the hydrophobic OxPt–IMDQ–C18 prodrug only induced minor tumor eradication *in vivo*. Possibly, the prodrug is insufficiently metabolized *in vivo*, likely fostered by limited aqueous solubility.

Conjugation of a drug to hydrophobic lipids is an established method to improve drug efficacy. Both the addition of fatty acid chains as well as cholesterol increase the levels of cellular uptake by different cell types. Indeed, we demonstrated in this work that the addition of a C18-lipid chain enhances cellular uptake of Pt prodrugs by cancer cells, as observed when comparing cellular Pt uptake by CT26 cells treated with IMDQ–OxPt–C18 and native OxPt, respectively. Furthermore, we confirmed that conjugation to PEG-cholesteryl and Pt–C18, respectively, decreased systemic inflammation of native IMDQ.

Amphiphile conjugation is an attractive strategy to improve aqueous drug solubility, alter the pharmacokinetic profile of the drug and increase cellular uptake by mediating interaction with the phospholipid plasma membrane. On the primary tumor, PEG-cholesteryl-conjugated OxPt and IMDQ could more potently reduce the tumor growth rate compared to the native drug molecule. Still, OxPt-PEG-cholesteryl monotherapy only reduced the tumor growth rate but did not result in complete tumor eradication. Combination therapy of OxPt-PEG-cholesteryl and IMDQ-PEG-cholesteryl treatment resulted in the formation of a hot tumor microenvironment and increased infiltration of anti-tumor leukocytes such as activated CD8+ T cells. Interestingly, IMDQ-PEG-cholesteryl monotherapy was already sufficiently potent to cure the primary tumor in 80% of the mice. We assume that IT injection causes local innate immune activation and likely also tumor cell death to some extent, resulting in recruitment of DCs that subsequently instigate an efficient anti-tumor response. Upon uptake by DC, IMDQ-PEG-cholesteryl enhances the antigen cross-presentation and activation of AH1 antigen-specific T cells in the tumor. We expect a lower anti-tumor response when IMDQ is administered systemically. This hypothesis is the subject of further research. PEG-cholesteryl conjugation to oxaliplatin and IMDQ did not interfere with the synergism observed between native oxaliplatin and IMDQ. The anti-tumor response on the primary tumor was similar between OxPt-PEG-cholesteryl + IMDQ-PEG-cholesteryl combination therapy or a combination of either of the native drugs.

The tumor-associated antigen (TAA) predominantly expressed in CT26 is GP70, which is processed into the AH1 epitope. Through cross-presentation of the antigen, DCs activate antigen-specific T cells. This adaptive anti-tumor immune response can provide protection against a rechallenge with CT26 tumor cells. Both OxPt and IMDQ-PEG-cholesteryl conjugates were superior compared to the native drugs in terms of mounting an anti-tumor response in the primary tumor. However, IMDQ-PEG-cholesteryl monotherapy only protected against a challenge tumor in 30% of the mice, while treatment with native IMDQ conferred a higher protection (*i.e.*, 83%). IMDQ-PEG-cholesteryl monotherapy mounted a local anti-tumor immune response, characterized by infiltration of CD45+ cells and a high ratio of AH1+ CD8+ T cells in the tumor, that was sufficient to eradicate primary tumors. We hypothesize that due to the more efficient uptake of the amphiphilic conjugates, the IMDQ could reach the cells in the lymph node and only locally innate immune activation occurred.

In summary, we describe a therapeutic approach to elicit antitumor immunity based on the intratumor administration of oxaliplatin and IMDQ. Our study underlines the synergism between oxaliplatin ICD-inducing chemotherapy and imidazoquinoline TLR7 agonist-mediated innate immune activation in cancer therapy. We propose conjugation to PEG-cholesteryl amphiphiles as an efficient strategy to decrease systemic distribution and increase the therapeutic effect on the primary tumor of both drugs while not interfering with their synergistic effects. A combination of OxPt-PEG-cholesteryl and IMDQ-PEG-cholesteryl successfully eliminated primary tumors and also mounted an adaptive immune response that protects against a tumor challenge and relapse.

## Materials and methods

### Cell lines

CT26 and 4T1 cells were obtained from ATCC. The Raw Blue reporter cell line, zeocin and Quanti Blue™ were obtained from InvVvoGen. CT26 (murine colon cancer cell line) cells and 4T1 (murine breast cancer cell line) cells were cultured in RPMI-Glutamax, supplemented with 10% FBS, 1 mM sodium pyruvate and 1% penicillin/streptomycin. Raw-Blue™ macrophages (murine macrophage reporter cell line) were cultured in DMEM, supplemented with 10% HI-FBS, 2 mM l-glutamine, 1 mM sodium pyruvate, 1% penicillin/streptomycin and 0.01% zeocin. Cells were incubated at 37 °C in a controlled, sterile environment of 95% relative humidity and 5% CO_2_.

### Materials and instrumentation

All chemicals were purchased from Sigma-Aldrich unless mentioned otherwise. Oxaliplatin was obtained from LC Laboratories. TLC plates were purchased from Macherey Nagel. Hydrogen peroxide 30% was obtained from VWR. IMDQ was provided to us by the David group and synthesized according to literature.^31^ Deuterated H_2_O (D_2_O) and DMSO-d6 were purchased from Deutero. Dulbecco's Modified Eagle Medium (DMEM), Roswell Park Memorial Institute 1640 Medium (RPMI 1640) – GlutaMAX™, fetal bovine serum (FBS), sodium pyruvate, penicillin, streptomycin, were obtained from Invitrogen.

All ^1^H- and ^13^C and 2D Nuclear Magnetic Resonance (NMR) spectra were recorded on a Bruker 300 MHz, 400 MHz or 500 MHz FT NMR spectrometer. Chemical shifts (*δ*) are provided in ppm relative to TMS. Samples were prepared in given deuterated solvents and their signals referenced to residual non-deuterated signals of the solvent. Electron spray ionization-mass spectroscopy (ESI-MS) was carried out on a Waters LCT Premier XETM TOF mass spectrometer with a ZsprayTM source and ESI and modular LocksprayTM interface, coupled to a Waters alliance HPLC system. UV-vis spectra were recorded on a Shimadzu UV-1650PC spectrophotometer. Spectra were always referenced to the dispersion medium prior to data collection. Cell culture plate readouts were collected on an Epoch 2 Microplate Spectrophotometer (Biotek, BioSPX, Drogenbos, Belgium). Mass cytometry measurements were conducted on a CyTOF2 mass cytometer (Fluidigm) and data were analyzed by FlowJo. Full body bioluminescence imaging was performed on a PerkinElmer Ivis Lumina system. Flow cytometry acquisition was performed with the BD FACSymphony A3 HTS (BD Biosciences).

### Synthesis of OXA(iv)–OH

The synthesis of the OXA-derivatives was performed according to modified procedures from earlier reports.^38–41^ Oxaliplatin (1.035 g, 2.61 mmol) was suspended in 30 mL of Milli-q water and 30% H_2_O_2_ (5 mL, 15.4 mmol) was added. The reaction mixture was stirred for 24 h, at room temperature in the dark. Next, the reaction mixture was condensed through rotary evaporation and the solid was re-dissolved in cold methanol and precipitated in cold diethyl ether. The precipitate was washed twice with cold diethyl ether and dried under vacuum to obtain OXA(iv)–OH as a white powder. Finally the chemical structure was characterized by NMR and ESI-MS (0.970 g, yield 87%). ^1^H-NMR (300 MHz, D_2_O/DMSO-d_6_): *δ* (ppm) 2.85–2.94 (m, 2H), 2.38 (m, 2H), 1.58–1.87 (m, 4H), 1.27–1.49 (m, 2H). ^13^C-NMR (75 MHz, DMSO-d_6_): *δ* (ppm) 166.21, 61.68, 31.68, 24.21. ESI-MS (negative ion mode) C_8_H_16_N_2_O_6_Pt: calculated [M − H]^−^ 430.058, [2 M − H]^−^ 861.124, found [M − H]^−^ 430.100, [2M − H]^−^ 861.100.

### Synthesis of Pt(iv)–COOH

In a second step, succinic anhydride (0.122 g, 1.22 mmol) was added to a solution of Pt(iv)–OH (0.500 g, 1.16 mmol) in 4 mL of DMSO. The reaction mixture was stirred for 24 hours at room temperature in the dark. Next, the solution was precipitated in diethyl ether, re-dissolved in methanol, and washed successively with cold acetone and diethyl ether. Finally the precipitate was dried *in vacuo* to obtain Pt(iv)–COOH as white solid. The chemical structure was characterized by NMR and ESI-MS (0.588 g, yield 95%).^1^H-NMR (300 MHz, DMSO-d_6_): *δ* (ppm) 12.01 (br. s, 1H), 8.28–8.57 (m, 1H), 7.97–8.26 (m, 1H), 7.69–7.95 (m, 1H), 6.88–7.34 (m, 1H), 2.52–2.61 (m, 2H), 2.30–2.47 (m, 4H), 1.99–2.13 (m, 2H), 1.39–1.57 (m, 3H), 1.30 (br. s., 1H), 1.04–1.22 (m, 2H). ^13^C-NMR (75 MHz, DMSO-d_6_): *δ* (ppm) 180.95, 174.06, 164.04, 61.46, 60.25, 31.65, 30.04, 23.89, 23.74. ESI-MS (negative ion mode) C_10_H_17_NO_4_: calculated [M − H]^−^ 530.074, [2 M − H]^−^ 1061.156, found [M − H]^−^ 530.100, [2M − H]^−^ 1061.100.

### Synthesis of Pt(iv)–C18

To a solution of Pt(iv)–COOH (0.350 g, 0.661 mmol) in 3 mL of anhydrous DMF, octadecyl isocyanate (0.204 g, 0.694 mmol) was added and stirred in the dark for 24 hours at room temperature. Next, the reaction mixture was filtered and DMF was removed under reduced pressure. The oily residue was then precipitated in cold diethyl ether (3 mL) and centrifuged. The solid was further washed twice with cold diethyl ether (10 mL) and cold dichloromethane (CH_2_CL_2_) (5 mL). After centrifugation, the yellow solid was dried *in vacuo* and characterized by NMR and ESI-MS (0.429 g, yield 78%).^1^H-NMR (DMSO-d_6_, 400 MHz): *δ* (ppm) 12.10 (br. s, 1H), 9.42–9.85 (m, NH), 8.52–8.83 (m, NH), 7.99–8.35 (m, 2 NH), 6.65–6.84 (m, NH), 2.89 (m, 2H), 2.56 (m, 2H), 2.39 (m, 2H), 2.09–2.22 (m, 2H), 1.45–1.56 (m, 2H), 1.04–1.43 (m, 36H), 0.85 (t, *J* = 6.71 Hz, 3H). ^13^C-NMR (75 MHz, DMSO-d_6_): *δ* (ppm) 179.32, 173.43, 164.20, 163.19, 60.92, 60.41, 31.07, 30.77, 30.66, 29.43, 29.34, 28.81, 28.61, 28.41, 26.07, 23.46, 23.20, 21.87. ESI-MS (negative ion mode) C_10_H_17_NO_4_: calculated [M − H]^−^ 826.369, found [M − H]^−^ 825.30.

### Synthesis of IMDQ–Pt(iv)–C18

Pt(iv)–C18 (0.070 g, 0.085 mmol) and HATU (0.034 g, 0.089 mmol) were dissolved in 1 mL anhydrous DMF. Next, triethylamine (TEA) (0.011 mg, 0.254 mmol) was added and stirred for 15 minutes at rt. Afterwards, IMDQ 2HCl (0.040 g, 0.093 mmol), dissolved in 0.5 mL anhydrous DMF, was added and the reaction mixture was mixed thoroughly. The reaction mixture was left to react overnight in the dark at room temperature. The next day, a saturated aqueous NaCl solution was added to the reaction mixture, resulting in precipitation of the compound. The precipitate was washed 3 times with demi-water and acetone. Finally, the product was dried *in vacuo* to provide an orange solid and further analysed by H-NMR and ESI-MS analysis (0.094 g, yield 97%). ^1^H NMR (400 MHz, DMSO-*d*_6_) *δ* ppm 8.00–8.43 (m, 4H), 7.91 (d, *J* = 7.90 Hz, 1H), 7.74 (d, *J* = 8.20 Hz, 1H), 7.50–7.58 (m, 1H), 7.25–7.32 (m, 1H), 7.18 (d, *J* = 8.06 Hz, 1H), 7.01 (d, *J* = 8.06 Hz, 1H), 6.68–6.79 (m, 1H), 5.90 (s, 2H), 4.12–4.27 (m, 1H), 2.94 (t, *J* = 7.30 Hz, 2H), 2.84–2.91 (m, 2H), 2.24–2.39 (m, 2H), 1.98–2.14 (m, 2H), 1.66–1.79 (m, 2H), 1.30–1.55 (m, 8H), 1.19–1.27 (m, 34H), 0.87 (t, *J* = 7.30 Hz, 3H), 0.85 (t, *J* = 6.40 Hz, 3H) ^13^C-NMR (75 MHz, DMSO-d_6_): *δ* (ppm) 180.49, 171.73, 163.99, 163.73, 154.04, 151.73, 144.65, 139.07, 135.16, 133.32, 127.85, 126.71, 126.52, 126.11, 125.12, 121.39, 120.36, 114.78, 61.20, 60.49, 41.69, 40.98, 31.58, 31.18, 31.10, 29.99, 29.71, 29.32, 29.12, 28.99, 26.59, 26.47, 23.95, 23.58, 22.36, 22.12, 14.02, 13.76 ESI-MS (positive ion mode) C_10_H_17_NO_4_: calculated [M + H]^+^ 1168.5769, [M + Na]^+^ 1190,5588, found [M + H]^+^ 1168.400, [M + Na]^+^ 1190.400.

### Synthesis of PEG-Pt(iv)–COOH

To a solution of Pt(iv)–COOH (0.060 g, 0.113 mmol) in 3 mL of anhydrous DMF, mPEG-isocyanate (0.258 g, 0.125 mmol) in 3 mL anhydrous DMF was added and stirred in the dark for 24 hours at room temperature. Next, DMF was removed under reduced pressure and the residue was precipitated in cold diethyl ether trice (30 mL) and centrifuged. After centrifugation, the yellow product was dried *in vacuo* and characterized by ^1^H-NMR and DOSY NMR (0.250 g, yield 85%).^1^H-NMR (300 MHz, MeOD): *δ* (ppm) 3.71–3.6 (s, 148H), 3.38 (s, 3H), 2.89–2.71 (m, 2H), 2.63–2.51 (m, 4H), 2.47–2.31 (m, 4H), 2.30–2.22 (m, 2H), 1.74–1.49 (m, 4H), 1.36–1.27 (m, 4H).

### Synthesis of PEG-Pt-Chol

PEG-Pt(iv)–COOH (0.150 g, 0.058 mmol) and HATU (0.022 g, 0.58 mmol) were dissolved in 2 mL anhydrous DMF. Next, triethylamine (TEA) (0.016 mg, 0.114 mmol) was added and stirred for 15 minutes at rt. Afterwards, cholesterol-amine (0.025 g, 0.063 mmol), dissolved in 1 mL anhydrous DMF, was added and the reaction mixture was mixed thoroughly. The reaction mixture was left to react overnight in the dark at room temperature. The next day, the crude was dialyzed against MeOH for multiple days and 1 day against water, followed by freeze drying. The white powder was further analyzed by ^1^H-NMR, ^13^C NMR and DOSY NMR (MeOD) (0.250 g, yield 85%).^1^H-NMR (300 MHz, CH_2_Cl_2_): *δ* (ppm): 5.42 (m, 1H), 3.85–3.79 (m, 2H), 3.71–3.6 (s, PEG H), 3.39 (s, 3H), 2.94 (m, 3H), 2.66–2.26 (m, 6H), 2.25–1.49 (m, 34H), 1.26 (s, 3H), 1.19–1.07 (m, 6H), 0.92 (d, *J* = 6.5 Hz, 3H), 0.87 (dd; *J* = 6.6 Hz, 1.9 Hz; 6H) 0.68 (s, 3H).

### *In vitro* cytotoxicity assay

The *in vitro* cytotoxicity profiles of the compounds against distinct cell lines (CT26, 4T1, and Raw Blue™ macrophages) were assessed by means of an MTT assay as described earlier.^42,43^ The MTT stock solution was prepared by dissolving 100 mg MTT in 20 mL of PBS and subsequent membrane filtration (0.220 μm). Before use, MTT stock solution was 5-fold diluted with culture medium. Stock solutions of Pt(iv) prodrugs were made in DMSO and dilution series in fresh medium were prepared. In brief, cells were seeded into 96-well titer plates (10^4^ − 5 × 10^4^ cells per well, suspended in 200 μL of culture medium) and incubated overnight. Next, cells were treated with 50 μL of fresh medium containing the test compounds at varying drug concentrations (final concentration range 0.1 to −10 μM), DMSO (positive control = 0% viability) or PBS (negative control = 100% viability), followed by either 24 h, 48 h or 96 h of incubation. Subsequently, the medium was aspirated and the cells were washed with 200 μL of PBS. After aspiration, 100 μL of MTT working solution was added and the cells were incubated for 2.5 h. Finally, the MTT working solution was aspirated and the formed purple formazan crystals were dissolved in 50 μL of DMSO. Absorbance was determined at 590 nm using an EnVision Multilabel plate reader. The absorbance of the positive control was used as a blank and therefore subtracted from all values. The dilution series, positive and negative control were added to the wells in quadruplicate (*n* = 4). Cell viability (%) was calculated according to the equation below.
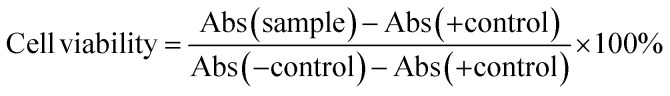


### *In vitro* cell stimulation assay

To evaluate the TLR activation potential of the IMDQ-modified prodrugs *in vitro*, a QUANTI-Blue™ assay was performed using RAW-Blue™ cells. Firstly, cells were seeded in a 96 well round bottom plate at a density of 90 000 cells per well (suspended in 200 μL). Next, cells were pulsed with 50 μL of fresh medium containing the test compounds at varying drug concentrations (final concentration range 0.1 to −10 μM), DMSO (positive control in MTT assay) or PBS (negative control) in fivefold. After 24 h of incubation at 37 °C, 50 μL of the supernatant was transferred into a 96 well flat bottom plate and incubated with 150 μL of Quanti blue solution at 37 °C, according to manufacturer's instructions (InvivoGen). The secreted embryonic alkaline phosphatase (SEAP) levels were determined by measuring the optical density at 620 nm using a microplate reader. Activity was determined by the increase in optical density relative to the negative control. To determine the *in vitro* cytotoxicity on RAW-Blue™ cells, the cell stimulation assay was combined with a MTT assay by using the abovementioned MTT protocol (vide 4.8.)

### Mass cytometry (CYTOF) analysis

CT26 cells (0.1 M mL^−1^) were seeded in a 6 well round bottom plate overnight (suspended in 2 mL). The next day, different drugs were added to the designed well at varying drug concentrations (2 μM, 5 μM and 10 μM) and incubated for 4 h or 24 h. Next, medium was discarded and cells were washed twice with PBS. Cells were then digested in the incubator for 3 min with 400 μL trypsin and further diluted to 1 M mL^−1^ for CyTOF staining. As life/dead stain, cells were treated for 15 min. at 37 °C with 500 μM Rhodium and then washed with PBS. After fixation and permeabilization, an iridium-containing intercalator (125 nM) was added for 20 min at room temperature for nuclear staining. Different drugs have been separated into 3 different batches, barcoded independently and measured separately. All conditions have been conducted in triplicate.

### *In vivo* bioluminescence imaging of innate immune activation

Heterozygous IFN-β-luciferase reporter mice (BALB/C), aged 7–9 weeks, were used for the experiment. Mice were housed in individual ventilated cages and given ad libitum access to food and water. Next, 50 μL of IMDQ, IMDQ–Pt(iv)–C18 and IMDQ-PEG-Chol were injected subcutaneously in the flank of the mice (*n* = 3) at an equivalent IMDQ dose. For *in vivo* imaging at the given time points, mice were injected subcutaneously with 150 mg per kg d-luciferin and *in vivo* luminescence imaging was recorded 12 min later using the IVIS Lumina II imaging system. Local luminescence and full-body luminescence were quantified using the Living Image 4.4 software. d-Luciferin potassium salt (15 mg mL^−1^ in PBS) was obtained from Gold Biotechnology, Inc (St. Louis, MO).

### Syngenic mouse model – intratumoral treatment and tumor challenge

Balb/c mice (6–8 weeks old) were injected subcutaneous with 5 × 10^5^ CT26 cells. 7 days after the vaccination treatment was administered *via* intratumoral injection in 50 μL solvent. Treatment concentration was calculated dependent on the body weight at time of treatment. Oxaliplatin 1.25 mg kg^−1^, IMDQ 1.13 mg kg^−1^, IMDQ–Pt(iv)–C18 3.68 mg kg^−1^, cisplatin 2.5 mg kg^−1^, IMDQ–Pt(iv) 2.88 mg kg^−1^, PEG-Pt(iv)-Chol 9.39 mg kg^−1^, PEG-IMDQ-Chol 12.49 mg kg^−1^. Tumors at the day of the treatment measured between 25 and 30 mm^3^. Tumor growth was measured every other day after treatment with an electronic caliper. Tumor volume was calculated as *V* = π × 1/6 × tumor length × tumor height × tumor width. Mice were preliminary sacrificed when the tumor reached a volume of 1000 mm^3^ or became necrotic.

Seven days after the treatment, at day 14 after tumor injection, mice were challenged on the opposite side by subcutaneous injection with 5 × 10^5^ untreated CT26 cells. Tumor growth on both sides was monitored every other day. During all *in vivo* experiments the researchers handling the mice were blind until after data analysis.

### Tetramer staining and IFN-γ production assay

Mice were injected subcutaneous with 5 × 10^5^ CT26 cells. 7 Days after the vaccination treatment was administered *via* intratumoral injection (cfr intratumoral treatment). 14 Days after treatment, day 21 since tumor inoculation, mice were sacrificed for collection of inguinal draining lymph nodes, spleen and tumor tissue. Weight of complete tumor and spleen tissue was measured directly after collection. Single cell suspension was prepared from the organs using 70 μM cell strainers. Spleen samples were treated with ACK lysis buffer (Lonza, 10-548E). Tumor tissue was minced and incubated for 1 h at 37 °C in with DNaseI (20 mg mL^−1^, Sigma-Aldrich, DN25) and collagenase IV (100 mg mL^−1^, Thermo Fisher, 17104019), after which leukocytes were isolated from the tumor tissue *via* percoll centrifugation. Some tumors were too small to collect a sufficient number of cells, therefore sample numbers can vary between followup of tumor growth and flow cytometry analysis. Lymphocytes from spleen, inguinal lymph node and tumor samples were pretreated to stimulate IFN-γ production in a 96 well plate with a T cell stimulation cocktail (eBioscience, 00-4975-93) for 4 h. Next cells were stained with MHC Dextramer (H-2 Ld/SPSYVYHQF-PE, Immudex, JG3294-PE), followed by anti-CD45-AF700 (eBiosciences, 56-0451-82), anti-CD19-PE-Cy5 (eBioscience, 15-0193-82), anti-CD3e-AF488 (BD Biosciences, 557666), anti-CD4-BUV737 (BD Bioscience, 612761), anti-CD8-eFluor450 (eBioscience, 48-0081-82) and fixable viability dye eFluor506 (eBioscience, 65-0866-18) in the presence of mouse Fc block (BD Biosciences, 553142). After overnight fixation and permeabilisation anti-IFNg-BV605 antibody (Biolegend, 505839) was added. Compensation was done with UltraComp eBeads (eBioscience, 01-2222-42). Flow cytometry acquisition was performed with the BD FACSymphony A3 HTS (BD Biosciences), data were analyzed using FlowJo software.

### Mice

Female WT BALB/c mice were ordered from Charles river Laboratories (France) and were housed in SPF conditions. Mice were 6–8 weeks old at the moment of experiment initiation and tumor injection. All experiments were evaluated and approved by the local Ethical Committee of Ghent University (EC2020-010). Sample size, number of mice, for each experiment was calculated *a priori* using G-power and is described in the EC-file. Within one experiment mice were randomly distributed over cages containing 6 mice that were all housed at identical conditions, treatments were administered simultaneously and *via* identical modes to control effect from confounders. Luciferase reporter mice (IFNβ+/Δβ-luc) with a BALB/c background were kindly provided by Prof. Johan Grooten (Department of Biomedical Molecular Biology, Ghent University).

### Statistics

Statistical analysis was performed in GraphPad Prism (v 9.3.0). Kaplan–Meier curves showing survival in the tumor models, complete recovery of the mice and onset of challenge tumor were analyzed by log-rank Mantel–Cox tests. Flow cytometry experiments, tumor volume, endpoint tumor size and spleen weight data were analyzed by one-way ANOVA with Tukey's post hoc test for multiple comparisons. These data are represented as mean ± SD unless indicated different. *p < 0.05, **p < 0.01, ***p < 0.001, ****p < 0.0001.

## Ethical approval

All mice were housed in specific pathogen-free conditions, and all experiments were performed according to guidelines of the local Ethics Committee of Ghent University. Experimental set-up for *in vivo* experiments was approved by the Ethics Committee of Ghent University (EC2020-010).

## Data availability

Data are available from the authors upon reasonable request.

## Conflicts of interest

There are no conflicts to declare.

## Supplementary Material

RA-015-D5RA00163C-s001
