# Supplementary material for: Enhancing anti-tumor immunity through intratumoral combination therapy with amphiphilic conjugates of oxaliplatin and imidazoquinoline TLR7/8 agonist
Source: RSC Adv. 2025 Apr 14;15(15):11662–74. doi: 10.1039/d5ra00163c (PMC11995270; doi:10.1039/d5ra00163c)
Supplement: RA-015-D5RA00163C-s001 [file RA-015-D5RA00163C-s001.pdf]

Supplementary Information (SI) for RSC Advances.
This journal is © The Royal Society of Chemistry 2025

Supporting Information

Enhancing Anti-Tumor Immunity through Intratumoral Combination Therapy with Amphiphilic Conjugates of Oxaliplatin and Imidazoquinoline TLR7 Agonist

Hanne Verschuere[1,2,3,#], Sabah Kasmi[3,4,#], Lutz Nuhn[5,6], Sènan D'almeida[7], Qiwen Zhu[8], Zifu Zhong[3,4], Sandy Adjemian[1,2,3], Benoit Louage[3,4], Jana De Vrieze[3,4], Haijun Yu[8], Bruno G. De Geest[3,4,*], Peter Vandenabeele[1,2,3,9,*]

[1] Cell Death and Inflammation Unit, VIB Center for Inflammation Research, Ghent, Belgium

[2] Department of Biomedical Molecular Biology (DBMB), Ghent University, Belgium

[3] Cancer Research Institute Ghent (CRIG), Ghent University, Belgium

[4] Department of Pharmaceutics, Ghent University, Ghent, Belgium

[5] Max Planck Institute for Polymer Research, Mainz, Germany

[6] Julius-Maximilians-Universität Würzburg, 97070 Würzburg, Germany

[7] CyTOF Flow Cytometry Core Facility, École Polytechnique Fédérale de Lausanne (EPFL), Lausanne, Switzerland

[8] State Key Laboratory of Drug Research & Center of Pharmaceutics, Shanghai Institute of Materia Medica, Chinese Academy of Sciences, Shanghai, 201203, China

[9] Methusalem program, Ghent University, Belgium

Share first authorship

* Share senior authorship

Corresponding authors: peter.vandenabeele@irc.vib-ugent.be, br.degeest@ugent.be

VIB-UGent Center for Inflammation Research, Technologiepark-Zwijnaarde 71, 9052 Ghent, Belgium

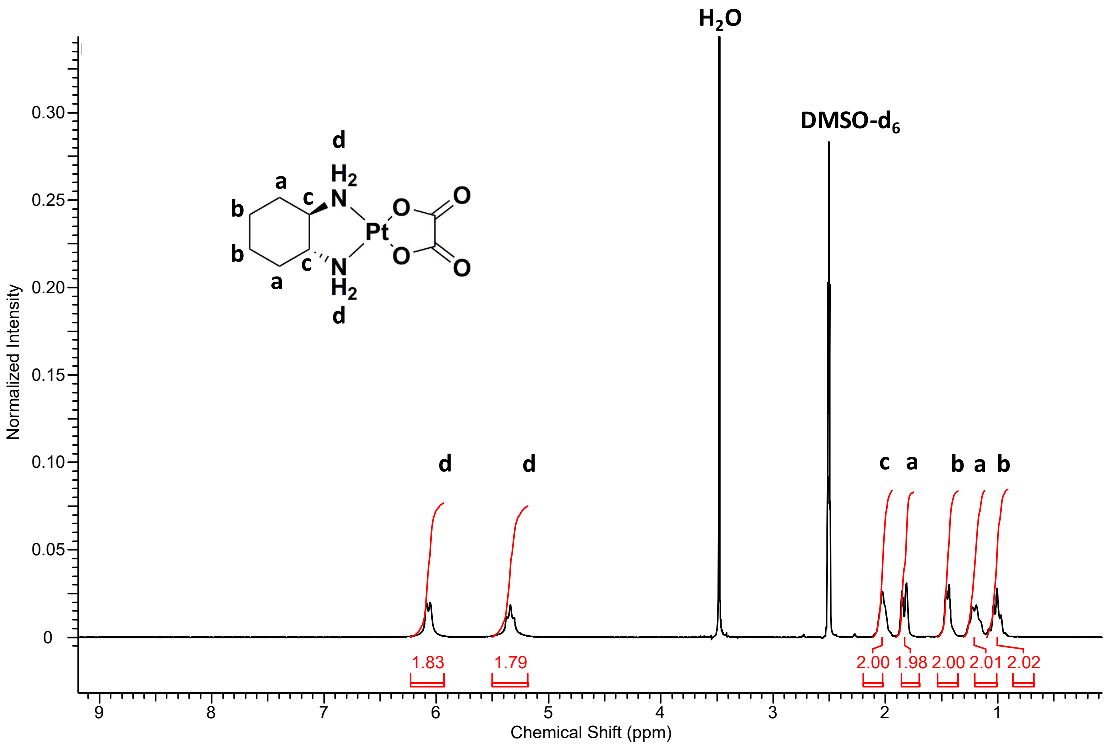

Figure S1. ^{1}H-NMR spectrum (300 MHz) of oxaliplatin in DMSO-d6.

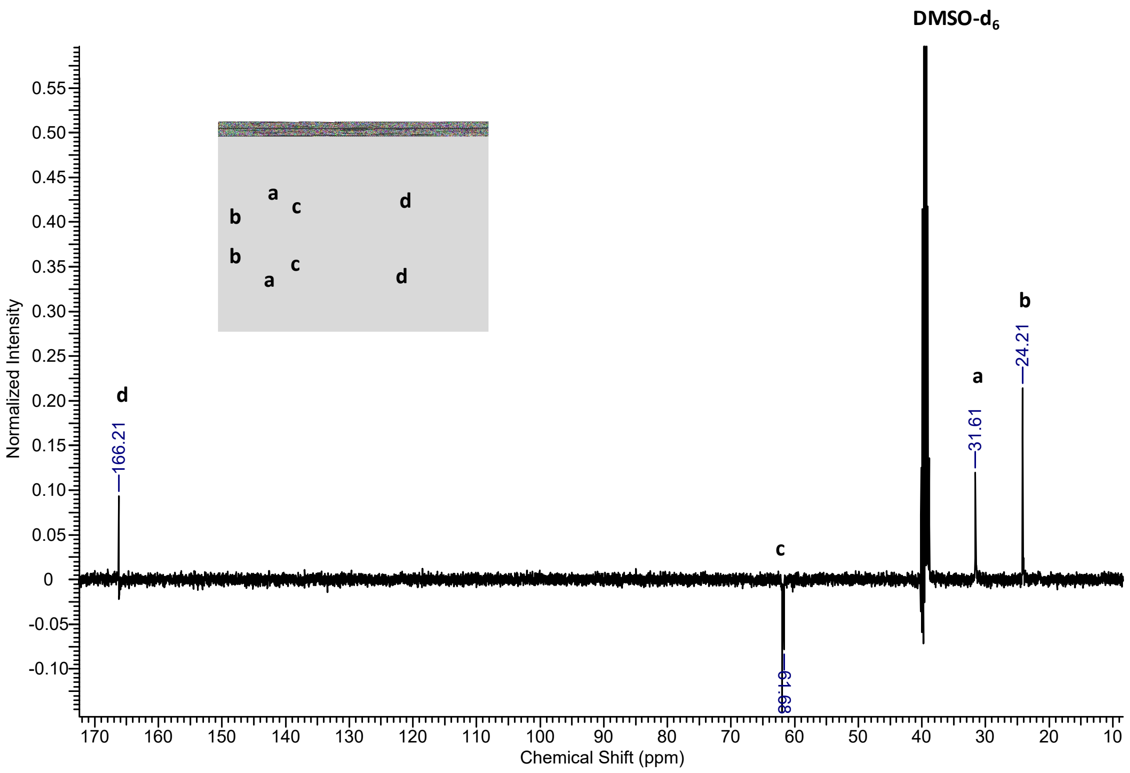

Figure S2. APT ^{13}C-NMR spectrum (75 MHz) of oxaliplatin DMSO-d6.

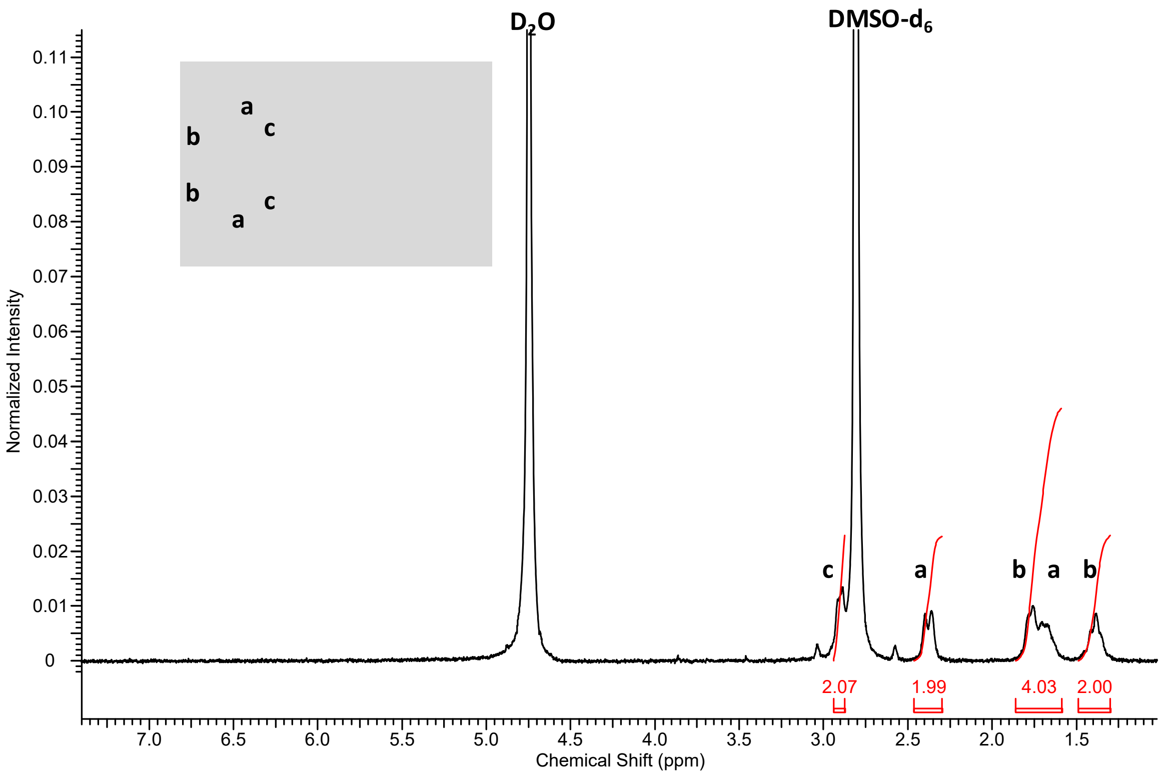

Figure S3. ^{1}H-NMR spectrum (300 MHz) of OxPt-OH in D_20/DMSO-d6.

.

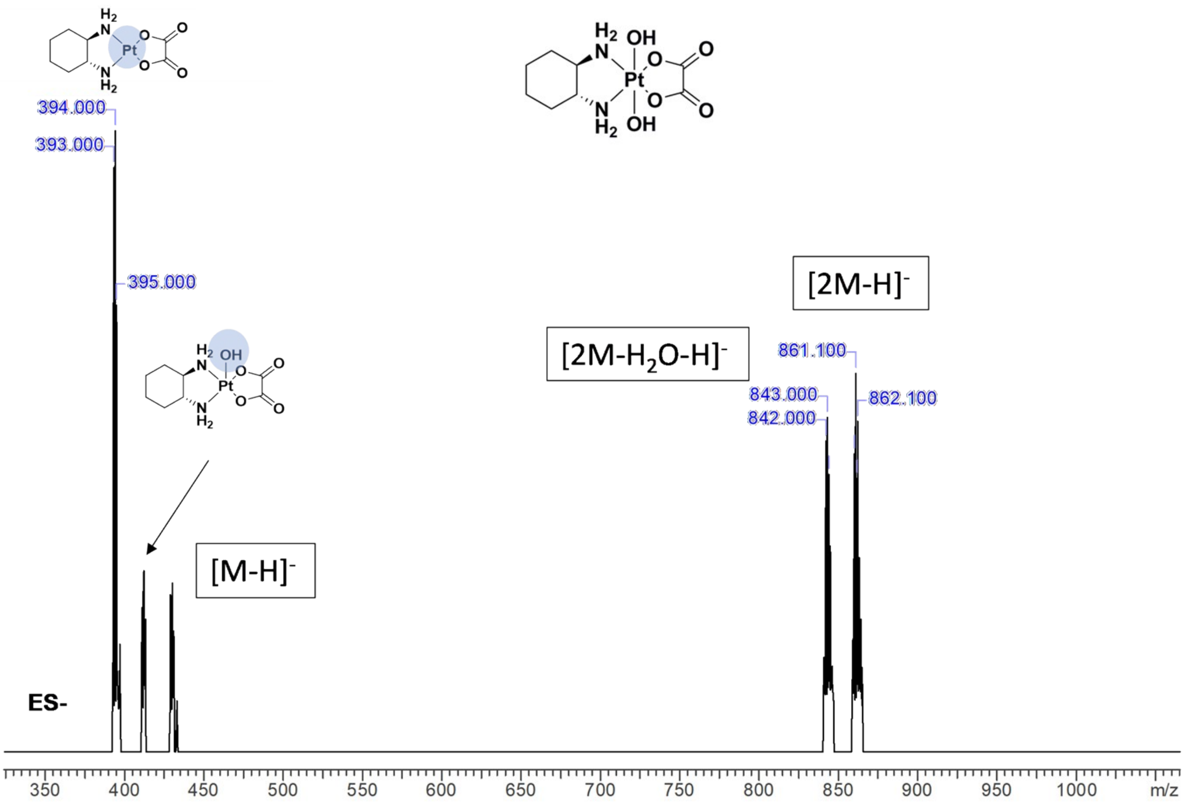

Figure S4. ESI-MS spectrum of OxPt-OH.

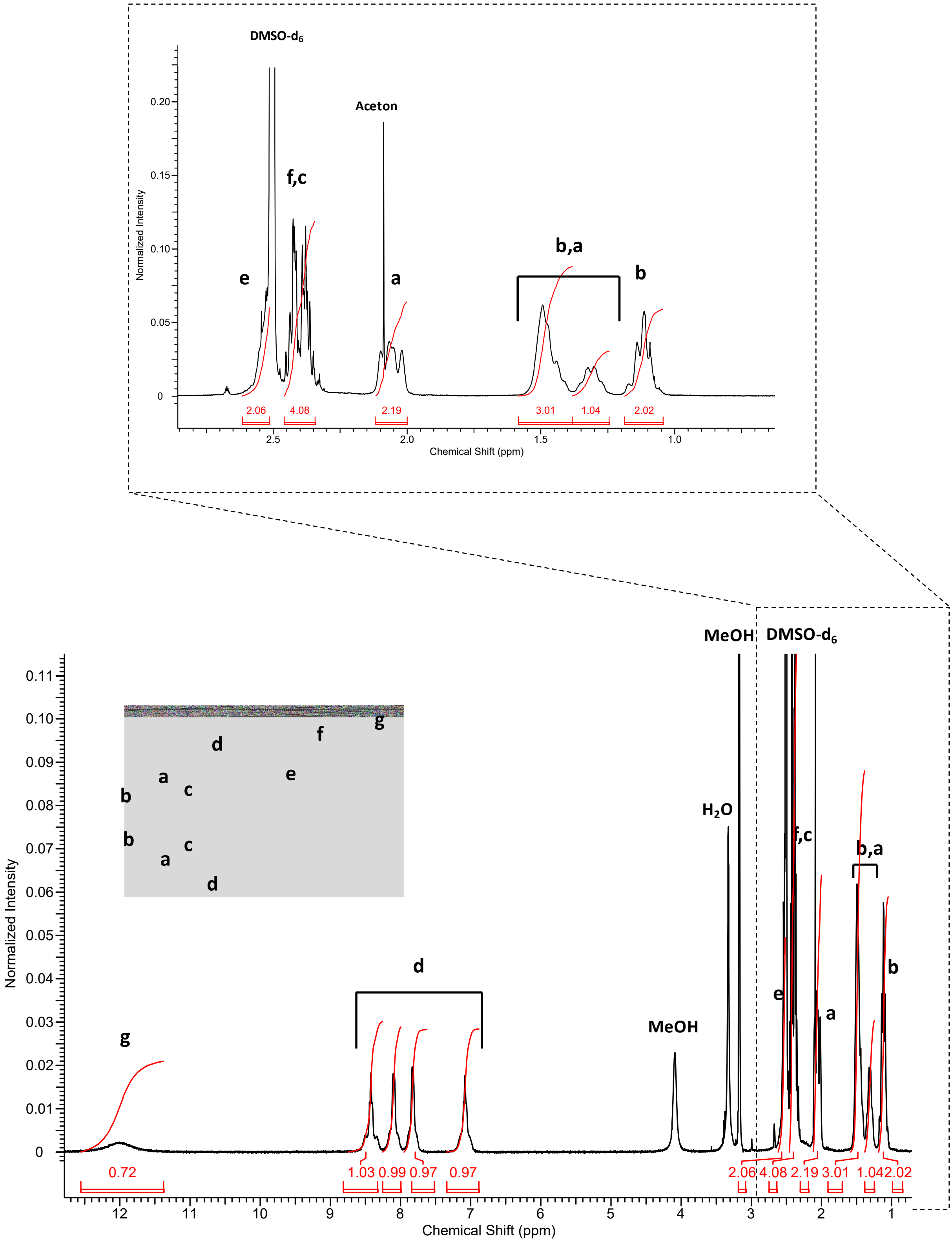

Figure S5. ^{1}H-NMR spectrum (300 MHz) of OxPt-COOH in DMSO-d_6.

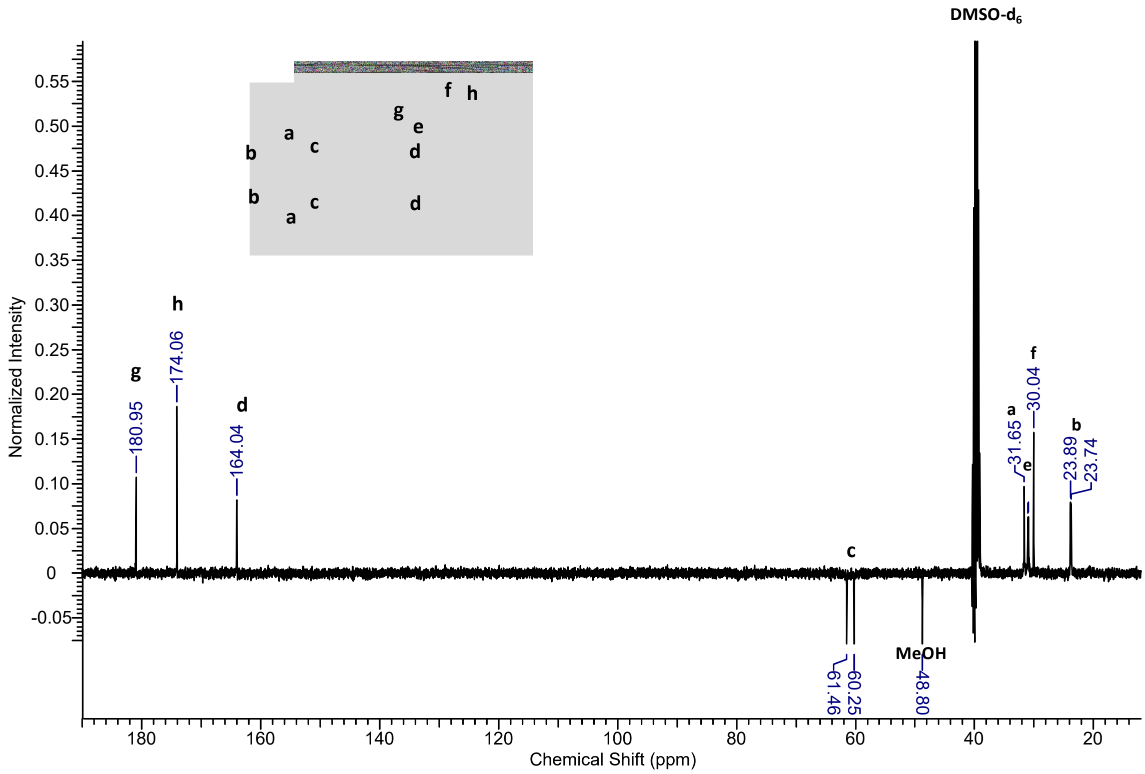

Figure S6. APT ^{13}C-NMR spectrum (75 MHz) of OxPt-COOH in DMSO-d_6.

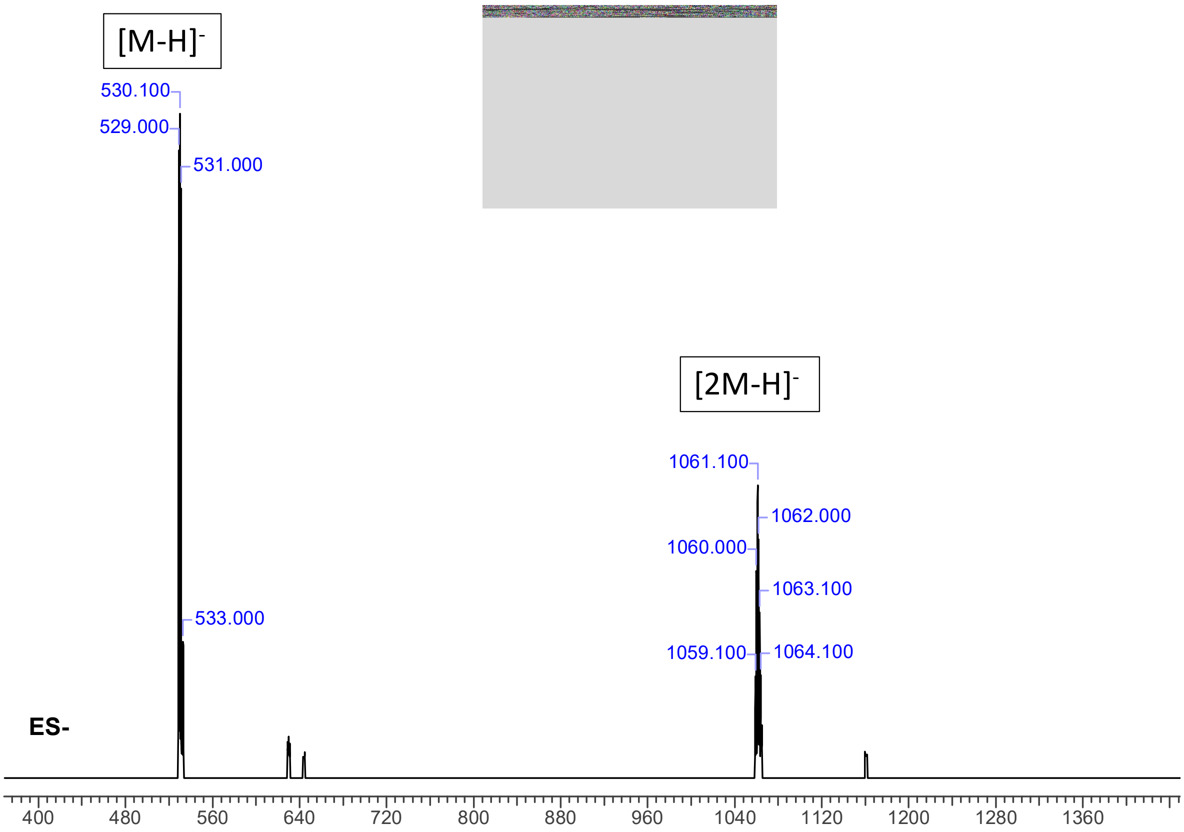

Figure S7. ESI-MS spectrum of OxPt-COOH.

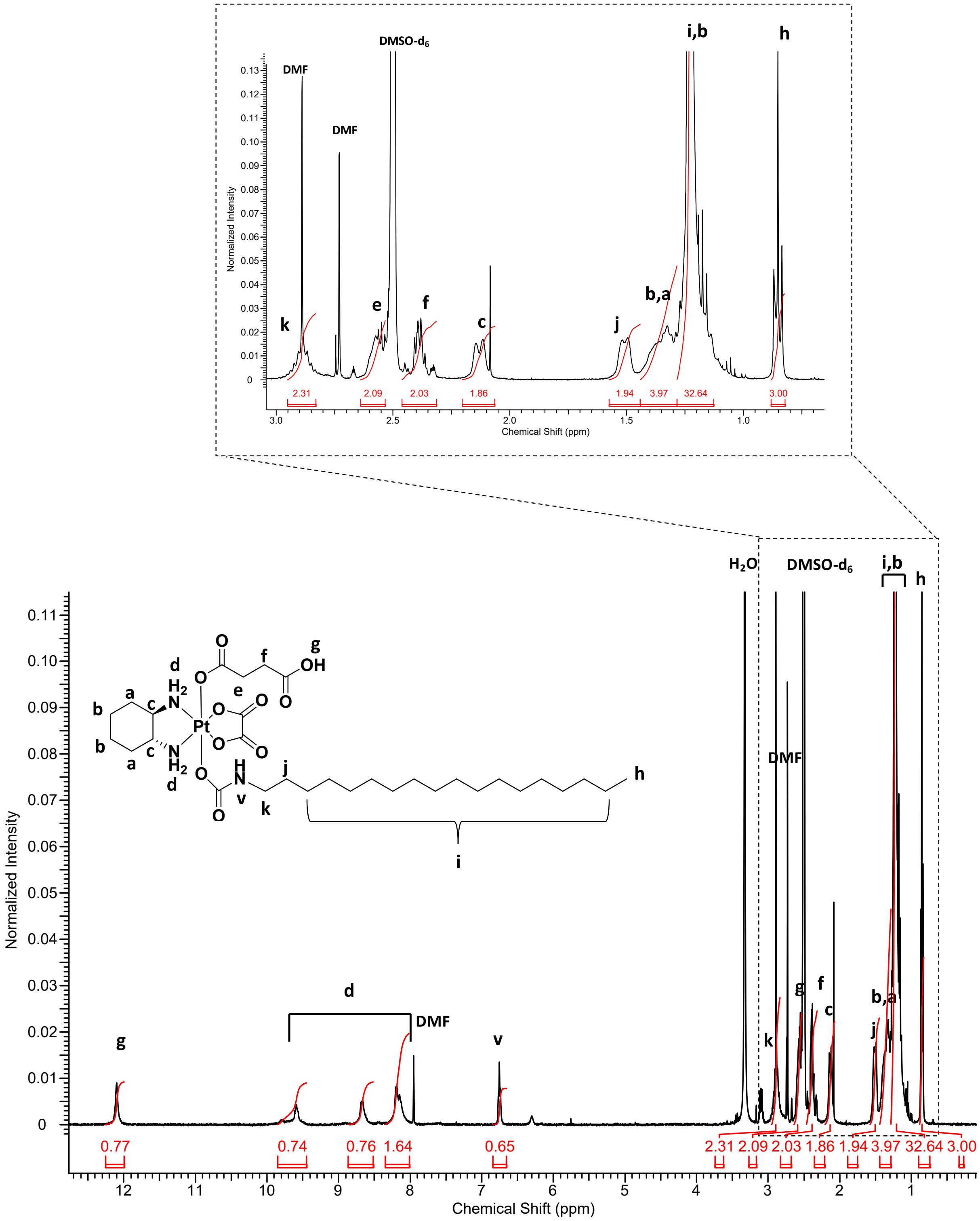

Figure S8. ^{1}H-NMR spectrum (300 MHz) of OxPt-C_{18} DMSO-d_6.

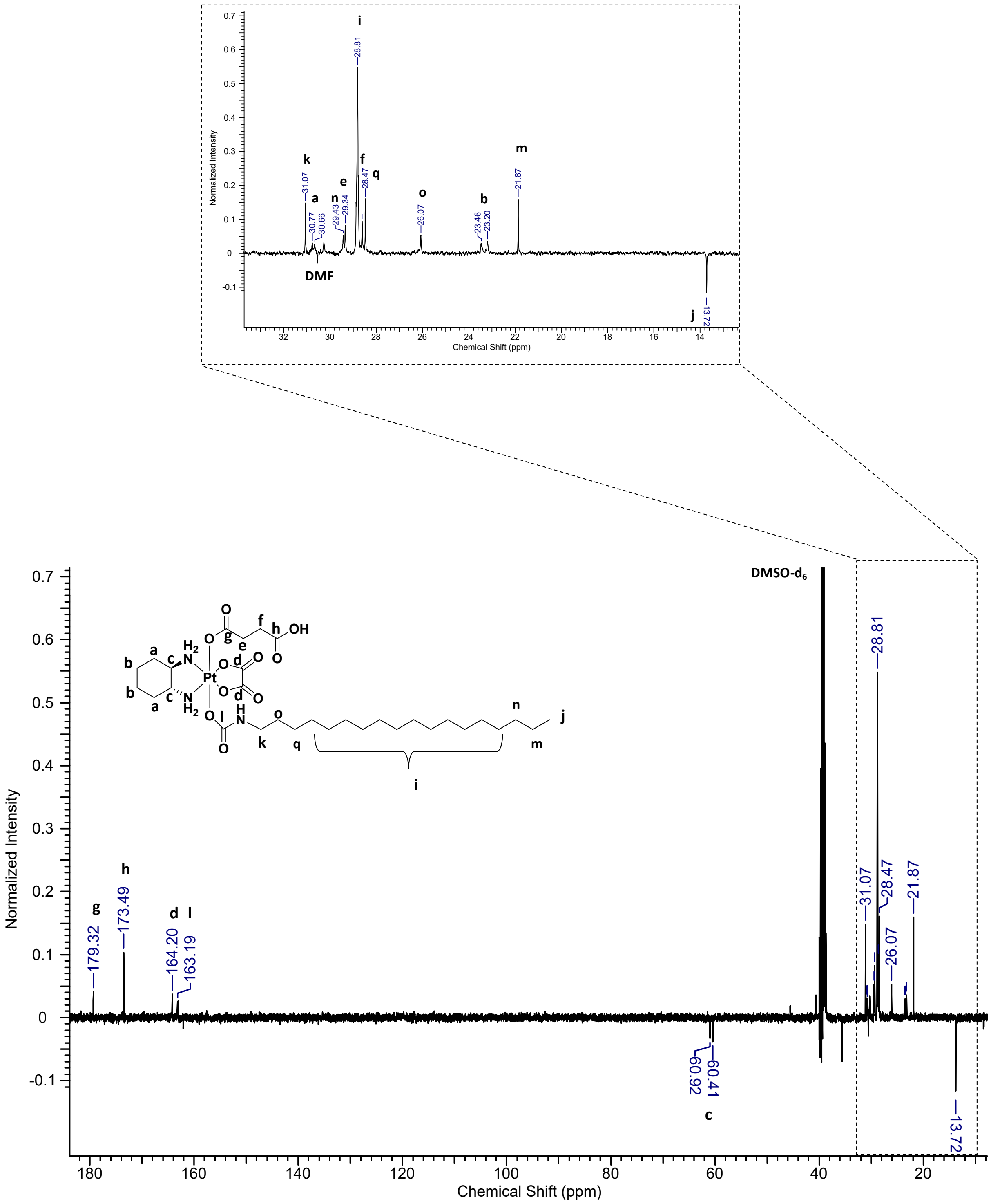

Figure S9. APT ^{13}C-NMR spectrum (75 MHz) of OxPt-C_{18} DMSO-d_6.

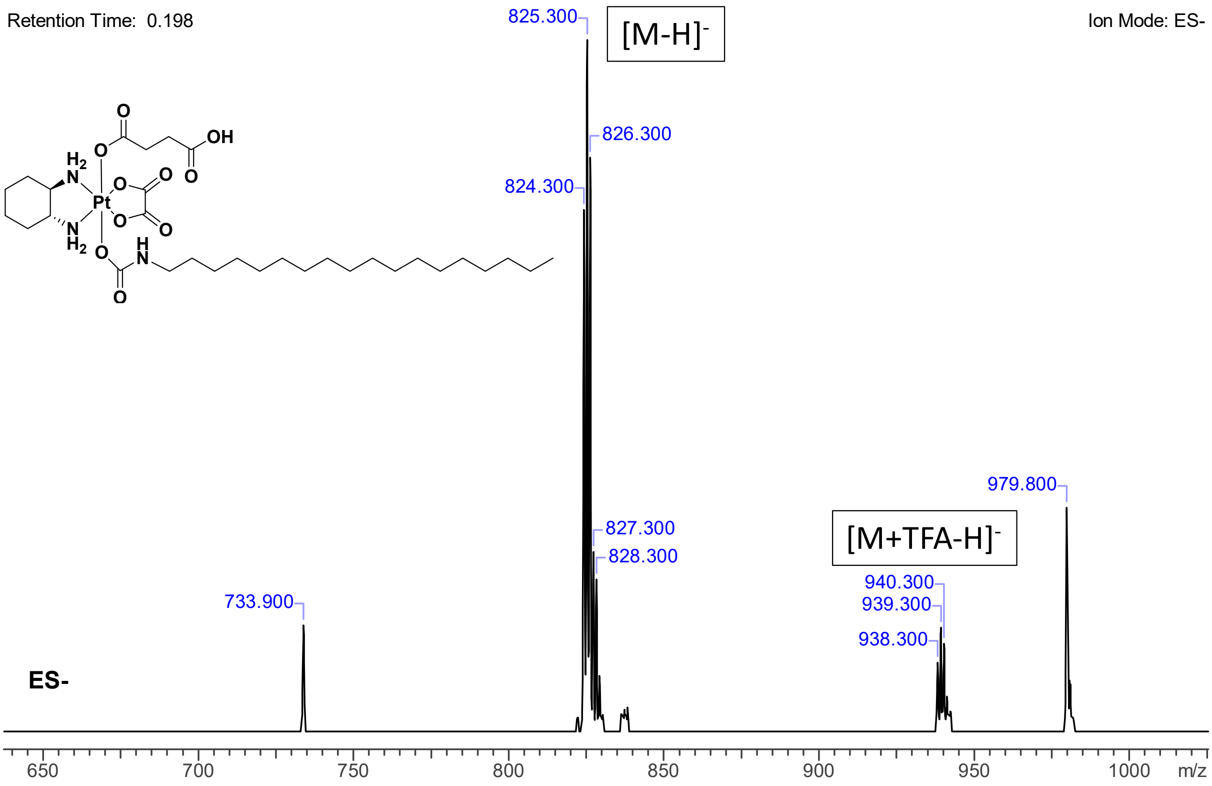

Figure S10. ESI-MS spectrum of OxPt-C_{18}.

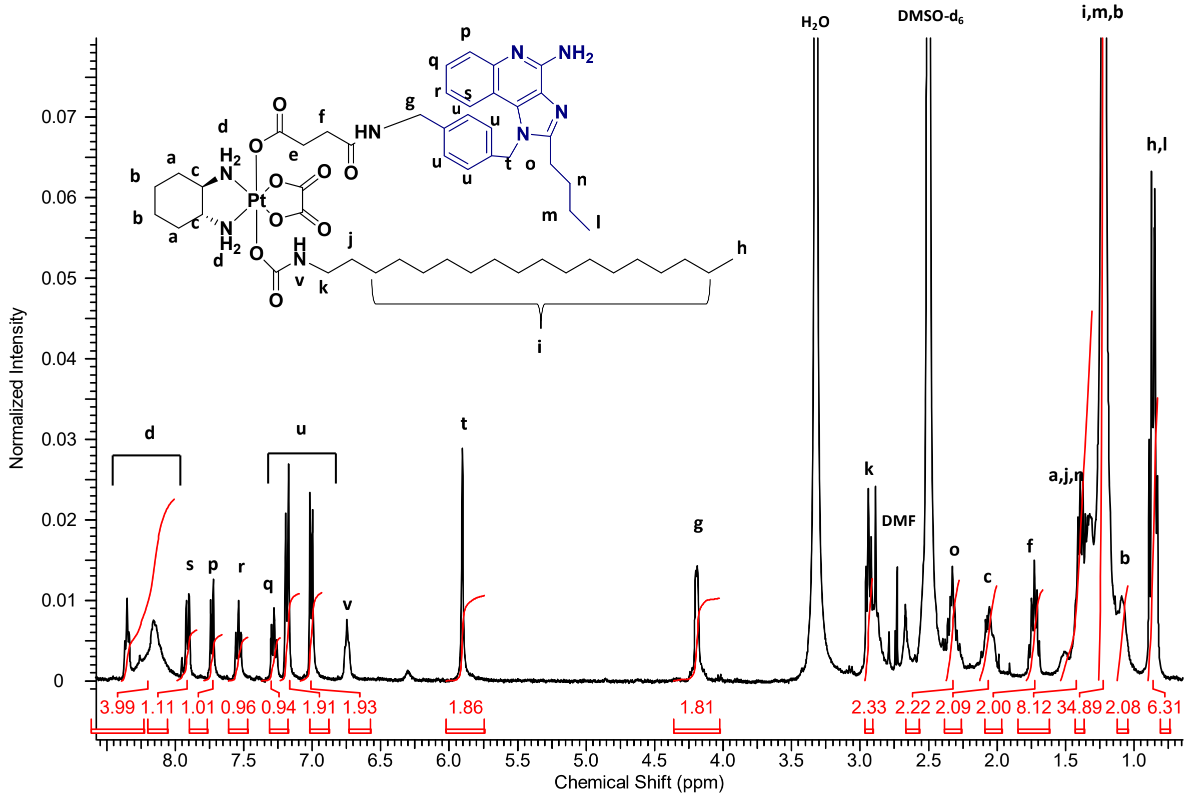

Figure S11. ^{1}H-NMR spectrum (300 MHz) of IMDQ-OxPt-C_{18} in DMSO-d_6.

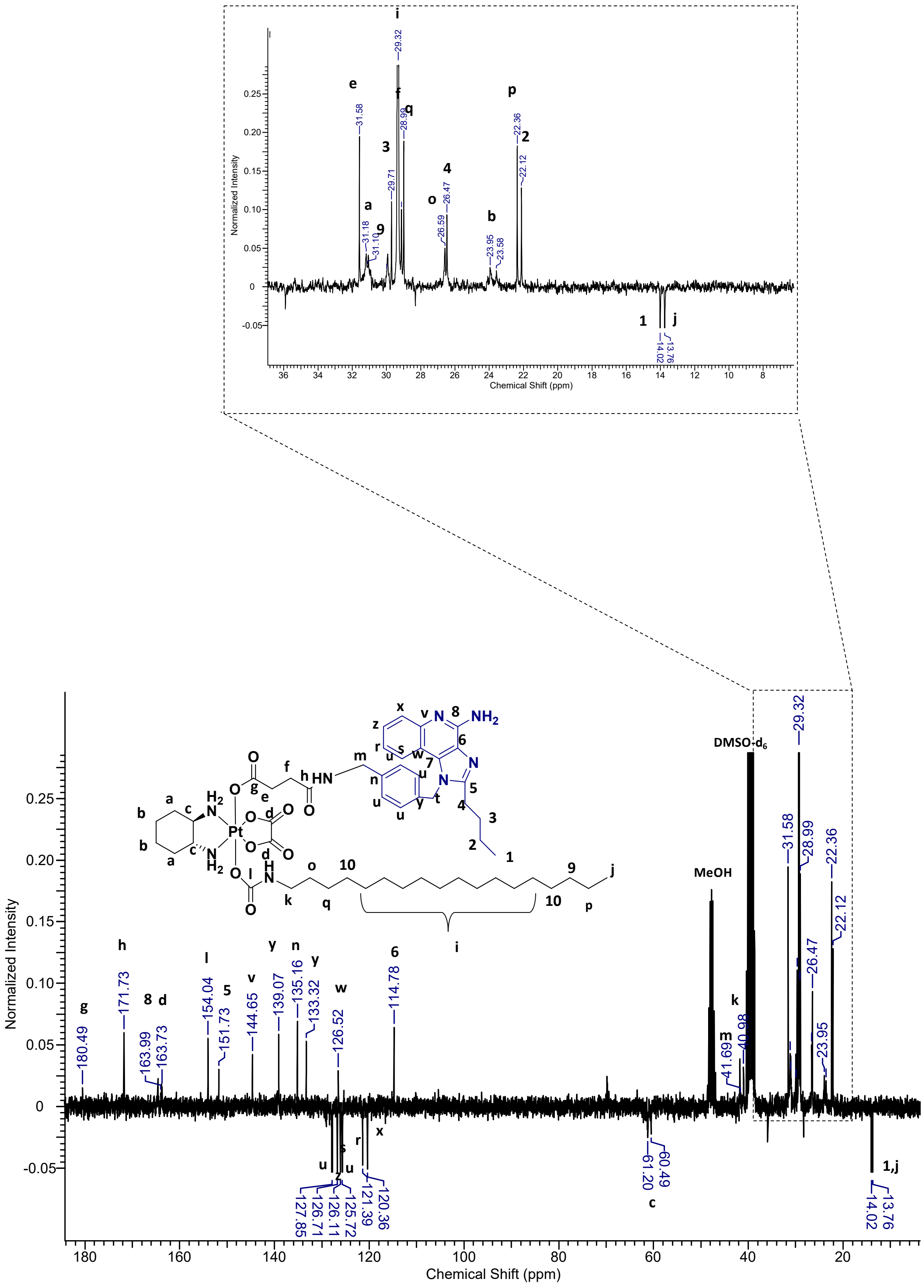

Figure S12. APT ^{13}C-NMR spectrum (75 MHz) of IMDQ-OxPt-C_{18} in DMSO-d_6.

Table 1. IC50 values of oxaliplatin and corresponding Pt (IV)-prodrugs after 96 hours on 3 cell lines.

	IC_{50} (µM)		
Cell line	Oxaliplatin (1)	Pt-C_{18} (4)	IMDQ-Pt-C_{18} (5)
CT26	1.10 ± 0.094	0.375 ± 0.024	0.697 ± 0.058

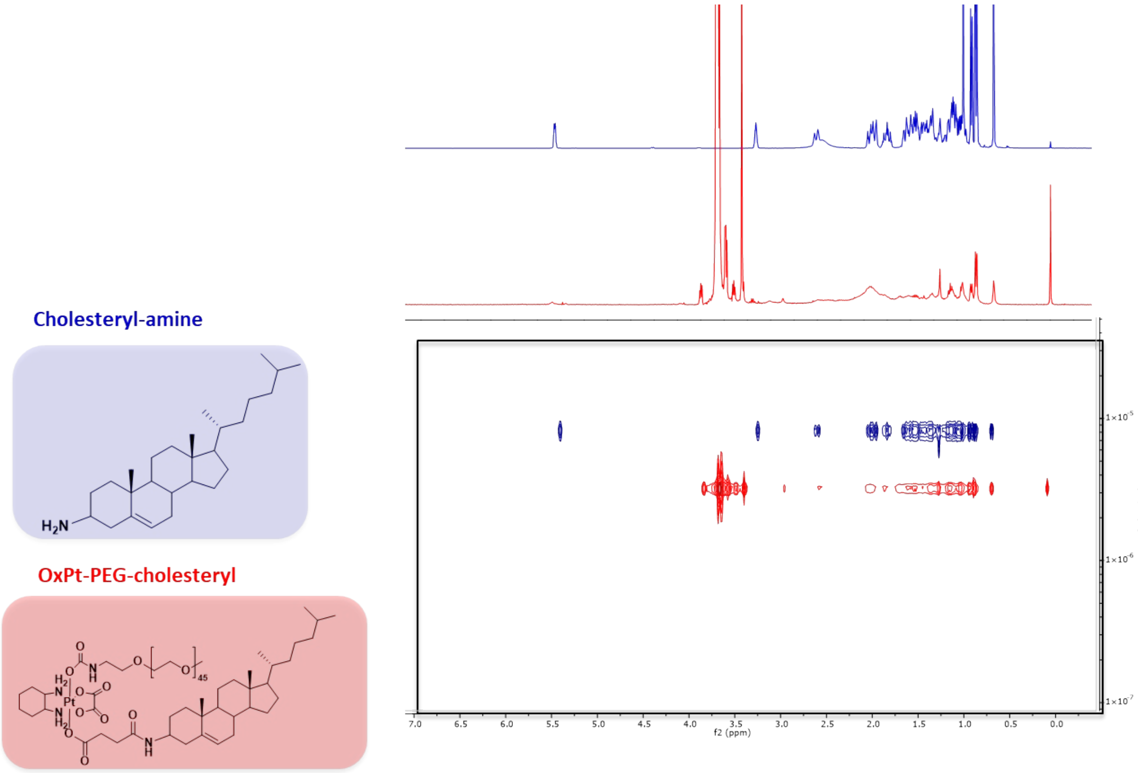

Figure S13. Overlay DOSY spectrum of OxPt-PEG-cholesteryl and cholesteryl-amine in $CDCl_3$ confirming successful conjugation of cholesteryl-amine to OxPt-PEG

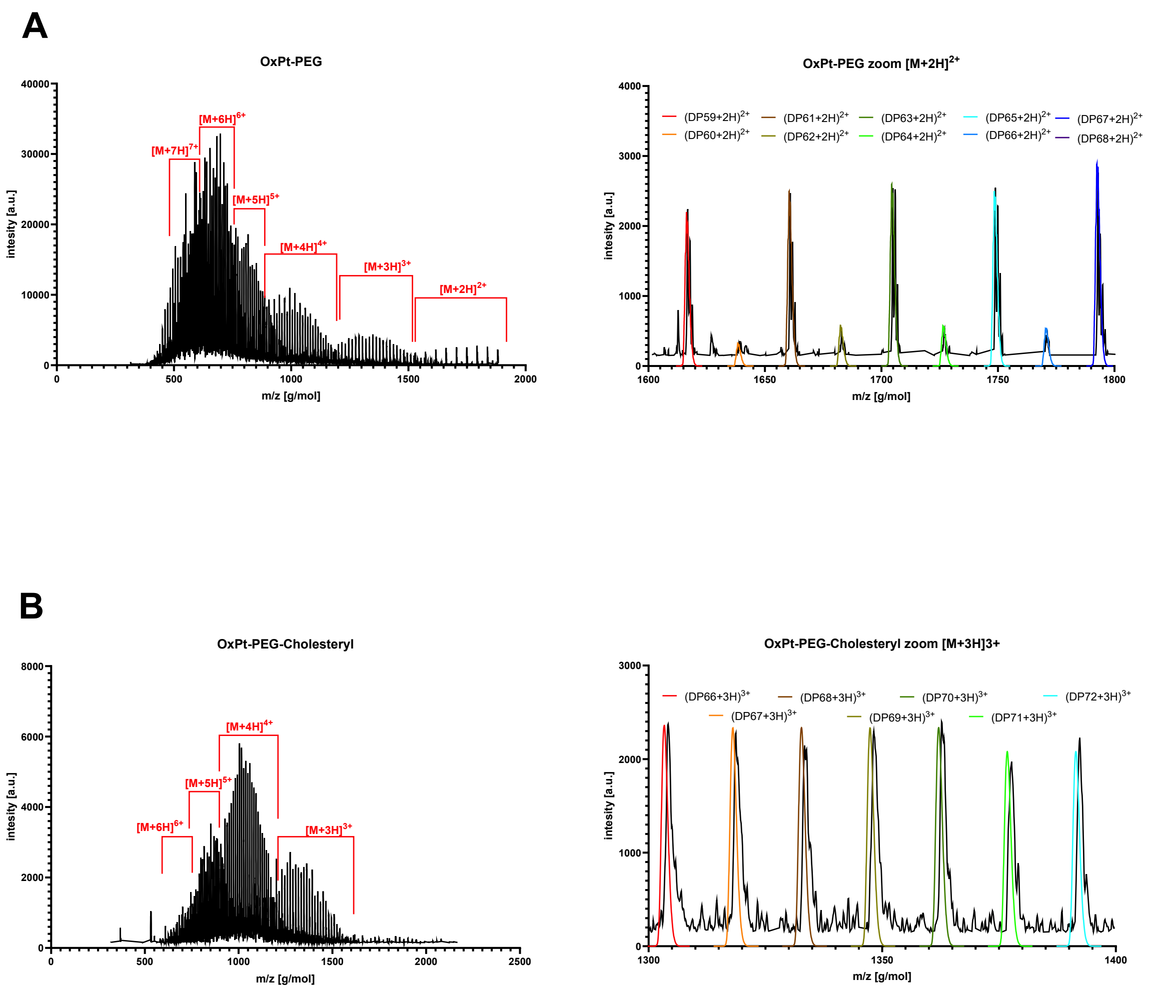

Figure S14. ESI-MS spectra of OxPT-PEG and OxPt-PEG-cholesteryl.

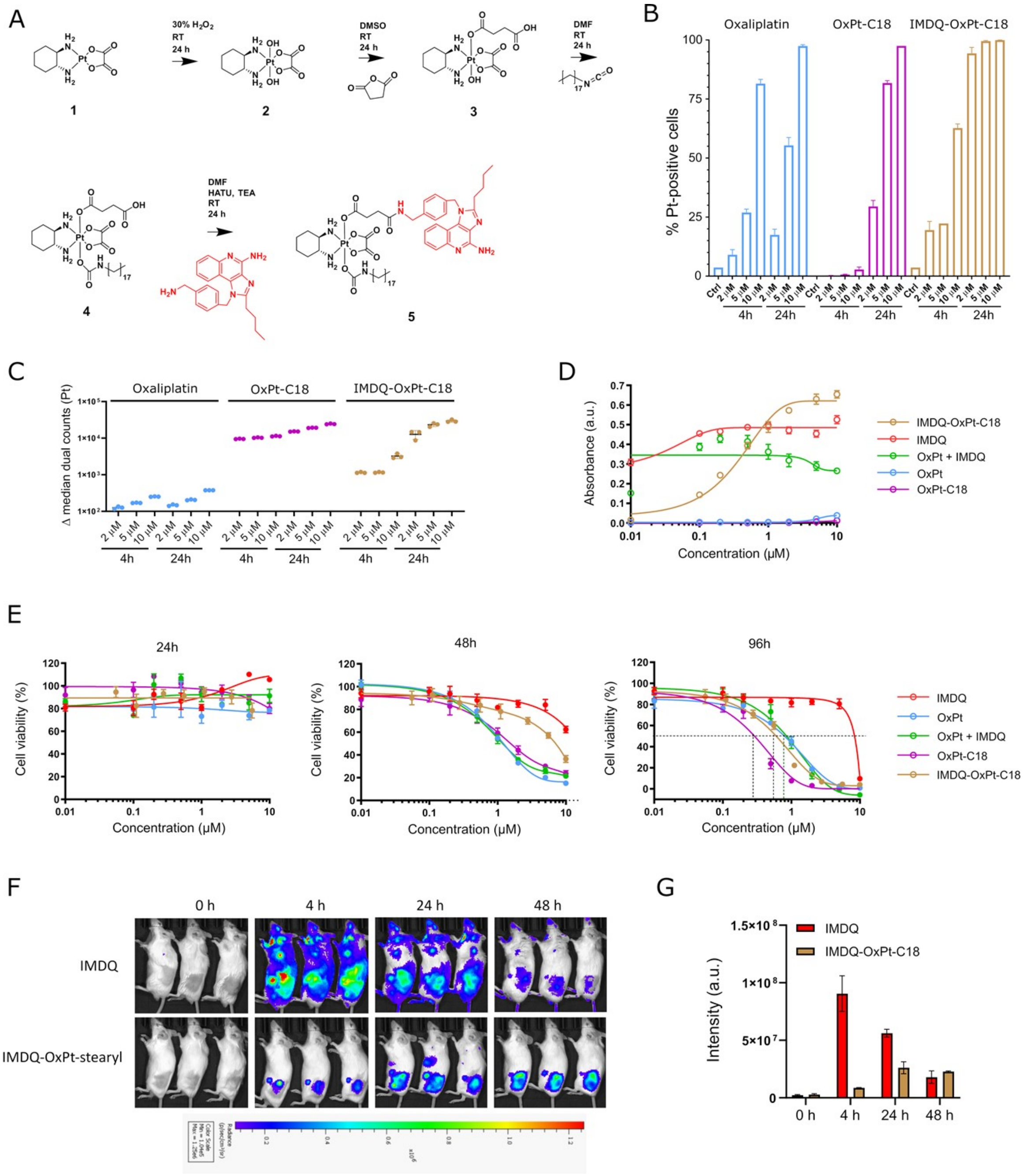

Figure S15. Synthesis, *in vitro* evaluation and bioluminescence imaging of IMDQ-OxPt-C18. (A) Synthesis route of IMDQ-OxPt-C18. **(B)** % of Pt-positive cells after 4 h and 24 h of incubation of CT26 cells with varying drug concentrations (2 µM, 5 µM and 10 µM) of soluble oxaliplatin or Pt(IV) conjugates. The cellular uptake of the platinum compounds was quantified by CyTOF mass cytometry *(n=3)*. Data represented as mean+SD. **(C)** Median of dual counts (MDC) of CT26 cells after incubation with either oxaliplatin or Pt(IV) compounds after 4 h and 24 h. Data represented as mean ±SD *(n=3)*. **(D)** TLR agonistic activity measured as NF-kB activation by a RAW-Blue reporter assay of the different compounds after 24 h of incubation *(n=5, mean+SD)*. **(E)** Cytotoxicity profiles of oxaliplatin and corresponding Pt(IV)-prodrugs after 24, 48 and 96 hours on CT26-cells, measured by MTT assay *(n=5, mean+SD)*. **(F)** *in vivo* innate immune activation of IMDQ and IMDQ-OxPt-C18 conjugate. Bioluminescence images of luciferase reporter mice (IFN-β$^{+/\Delta\beta\text{-luc}}$ mice; images taken 4 h, 24 h and 48 h

after subcutaneous injection of native IMDQ and IMDQ-OxPt-C18 in the flank. **(G)** Quantification of total luciferase luminescence intensity.

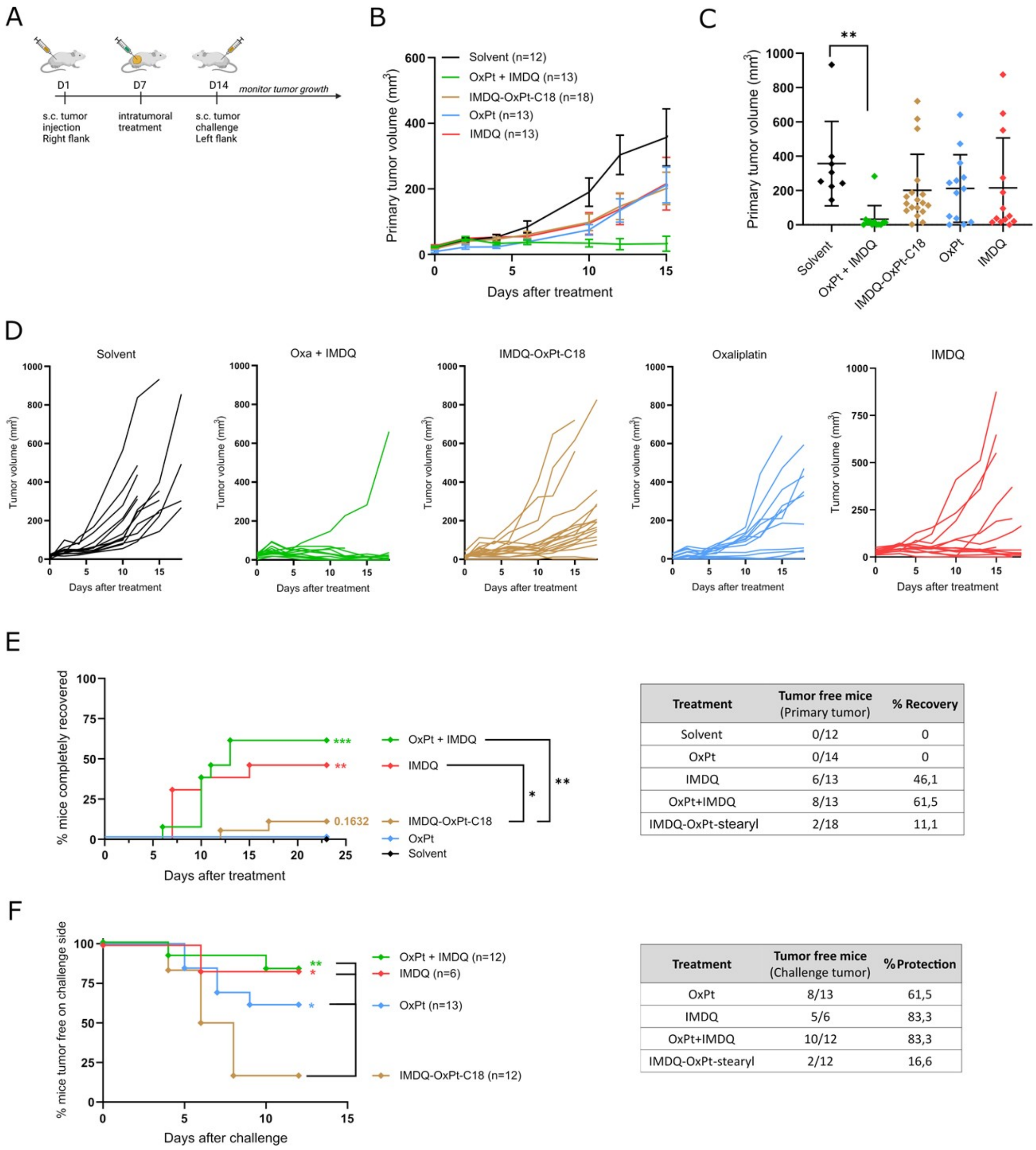

Treatment	Tumor free mice (Primary tumor)	% Recovery
Solvent	0/12	0
OxPt	0/14	0
IMDQ	6/13	46,1
OxPt+IMDQ	8/13	61,5
IMDQ-OxPt-stearyl	2/18	11,1

Treatment	Tumor free mice (Challenge tumor)	%Protection
OxPt	8/13	61,5
IMDQ	5/6	83,3
OxPt+IMDQ	10/12	83,3
IMDQ-OxPt-stearyl	2/12	16,6

Figure S16. Evaluation of intratumoral IMDQ-OxPt-C18 therapy. (A) Visualization of the experimental setup. Balb/c mice were injected subcutaneous with 5 x 10^5 CT26 cells to start tumor formation. After 7 days tumors were treated intratumorally (IT). Another 7 days later 5 x 10^5 CT26 cells were injected in the opposite flank to induce a challenge tumor. *(Solvent n=12; OxPt+IMDQ n=13; IMDQ-OxPt-C18 n=18; IMDQ n=13; OxPt n=14 for all subfigures except F, pool from 3 independent experimental repeats)* **(B)** Volume of the primary subcutaneous tumors measured from the moment of IT treatment. Data represented as mean ±SD. **(C)** Tumor volume at day 15 after treatment. Data represented as mean±SD, dots depict different mice. All significant comparisons are indicated. **(D)** Tumor growth progression over time starting at the moment of treatment, each mice is visualized individually until the moment of euthanasia. **(E)** Complete tumor recovery. When tumor size of the primary tumor decreased below 25 mm^3 and only scar tissue was present this was considered complete recovery. Asterisks next to the

curve indicate significant differences with solvent. Statistic in the legend compares different treatments. Absolute numbers are given in the table. **(F)** Registration of the onset of challenge tumors. Graph starting from the moment of challenge, on day 14 of the total experiment. Mice that did not form a challenge tumor were considered tumor-free on the challenge side. Statistics indicate comparison with IMDQ-OxPt-C18. Absolute numbers are given in the table. **p<0.05, **p<0.01, ***p<0.001, ****p<0.0001.*

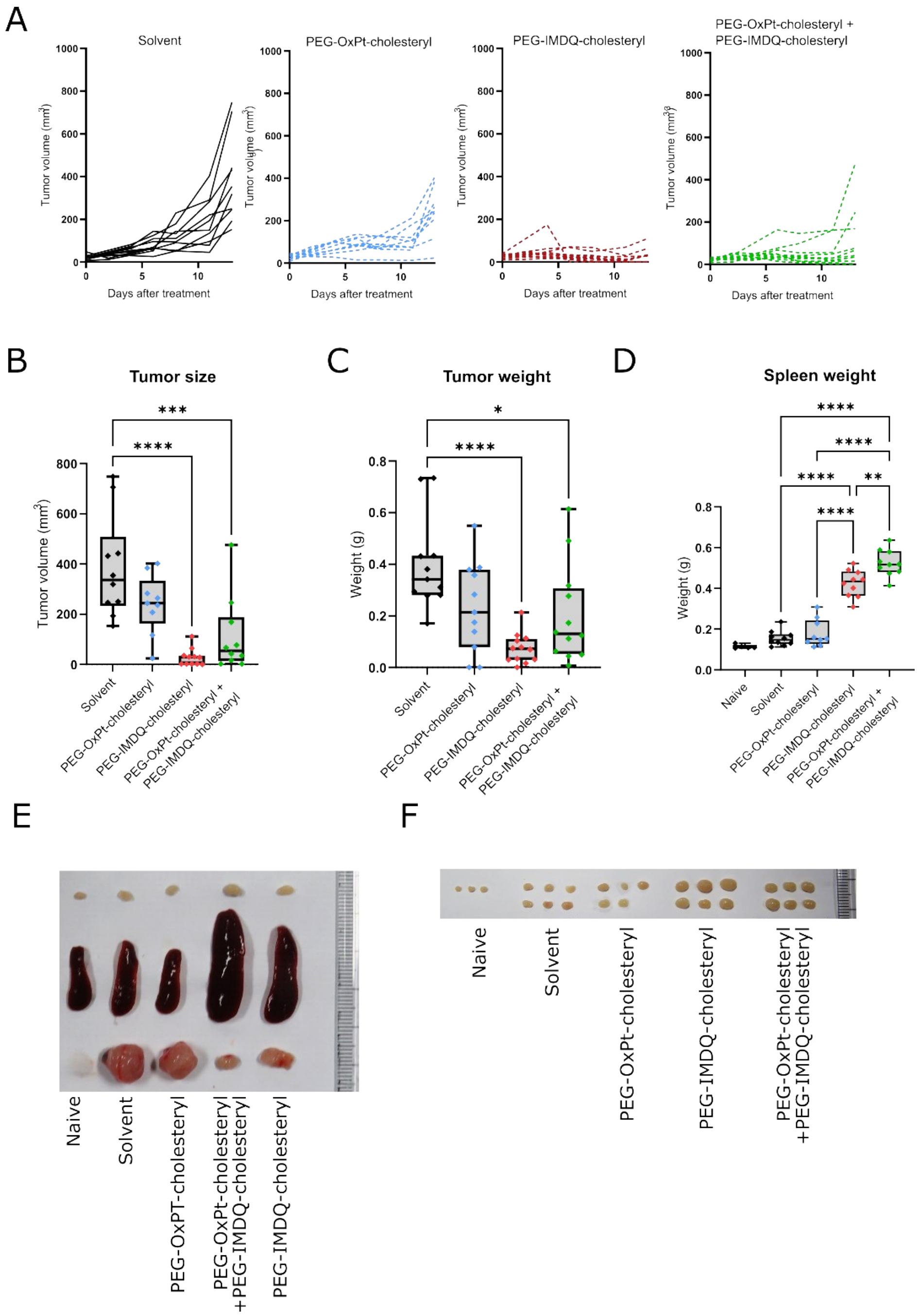

Figure S17. (A) Volume of the subcutaneous tumors measured from the moment of treatment, each mouse is visualized individually until the moment of euthanasia until day 15 (Solvent n=10, PEG-OxPt-cholesteryl n=9, PEG-IMDQ-cholesteryl n=11, PEG-OxPt-cholesteryl+PEG-IMDQ-cholesteryl n=10). (B) Tumor volume and (C) tumor weight at moment of sacrifice. Each dot depicts a separate mouse. All significant differences are indicated. (D) Weight of the spleen varies between different treatments. (E)

Representative samples of spleen, tumor draining lymph node and tumor tissue for each treatment at moment of sacrifice. F Representative picture of inguinal draining lymph nodes after different treatments. *$p<0.05$, **$p<0.01$, ***$p<0.001$, ****$p<0.0001$*

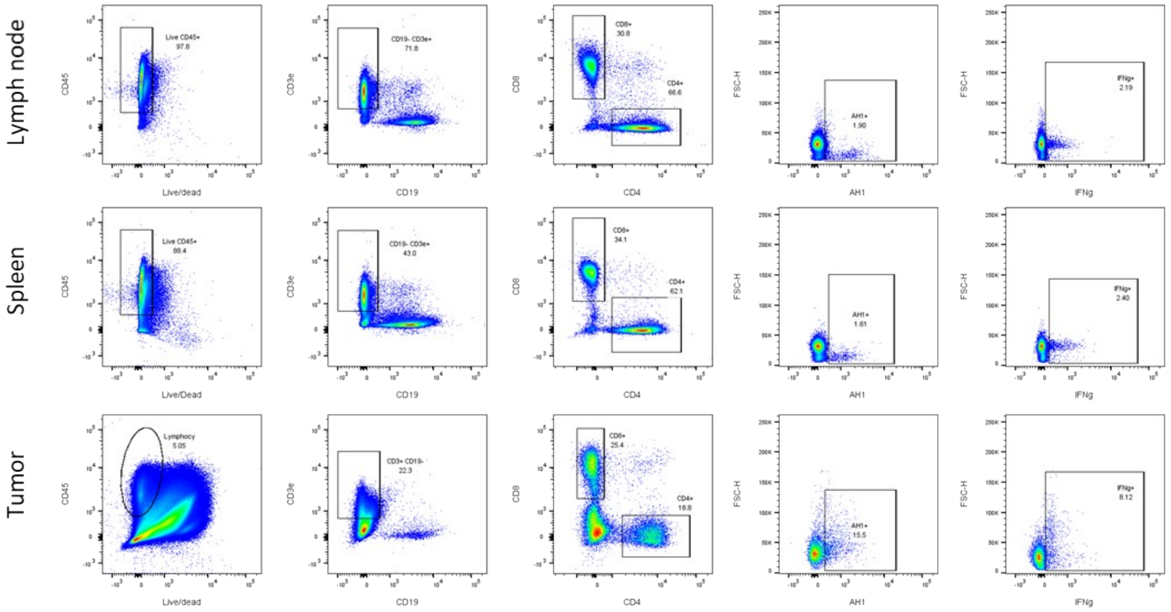

Figure S18. Gating strategy to select CD8/CD4 lymphocytes. First, live CD45+ cells were selected. From this population CD3e+/CD19- were identified. This subpopulation now contains T cells which can be divided in either CD8+ or CD4+. In each specific population the % of cells expressing AH1 specific MHC complexes or the cells producing IFNγ were identified.
